# Using genetics to examine the overall and sex-specific associations of branch-chain amino acids and the valine metabolite, 3-hydroxyisobutyrate, with ischemic heart disease and diabetes: a two-sample Mendelian randomization study

**DOI:** 10.1016/j.atherosclerosis.2023.117246

**Published:** 2023-08-21

**Authors:** Jie V Zhao, Bohan Fan, Stephen Burgess

**Affiliations:** 1 School of Public Health, Li Ka Shing Faculty of Medicine, The University of Hong Kong, Hong Kong SAR, China; 2 Medical Research Council Biostatistics Unit, University of Cambridge, UK; 3 Cardiovascular Epidemiology Unit, Department of Public Health and Primary Care, University of Cambridge, UK

**Keywords:** branch-chain amino acids, 3-hydroxyisobutyrate, ischemic heart disease, diabetes, Mendelian randomization, sex-specific

## Abstract

**Background and aims:**

Branch-chain amino acids (BCAAs) are linked to higher risk of diabetes, whilst the evidence on ischemic heart disease (IHD) is limited. Valine metabolite, 3-hydroxyisobutyrate (3-HIB), also plays an important role in metabolism, whilst its effect has been rarely examined. At the situation of no evidence from large trials, we assessed the role of BCAAs and 3-HIB in IHD and diabetes using Mendelian randomization to minimize confounding. Given their potential role in sex hormones, we also examined sex-specific associations.

**Methods:**

We used genetic variants to predict BCAAs and 3-HIB, and obtained their associations with IHD and diabetes in large consortia and cohorts, as well as sex-specific association in the UK Biobank and DIAGRAM. We obtained and combined the Wald estimates using inverse variance weighting, and different analytic methods robust to pleiotropy.

**Results:**

Genetically predicted BCAAs were associated with higher risk of IHD (odds ratio (OR) 1.19 per standard deviation (SD) increase in BCAAs, 95% confidence interval (CI) 1.05 to 1.35) and diabetes (OR 1.20, 95% CI 1.08 to 1.34). The associations with IHD were stronger in women (OR 1.23, 95% CI 1.03 to 1.48) than men (OR 0.96, 95% CI 0.83 to 1.10). 3-HIB was associated with higher risk of IHD (OR 1.43, 95% CI 1.17 to 1.73) but not diabetes, with no sex disparity.

**Conclusion:**

BCAAs and 3-HIB are potential targets for prevention in IHD and/or diabetes. BCAAs may exert a sex-specific role in IHD. Consideration of the sex disparity and exploration of the underlying pathways would be worthwhile.

## Graphical abstract

**Figure 4:**
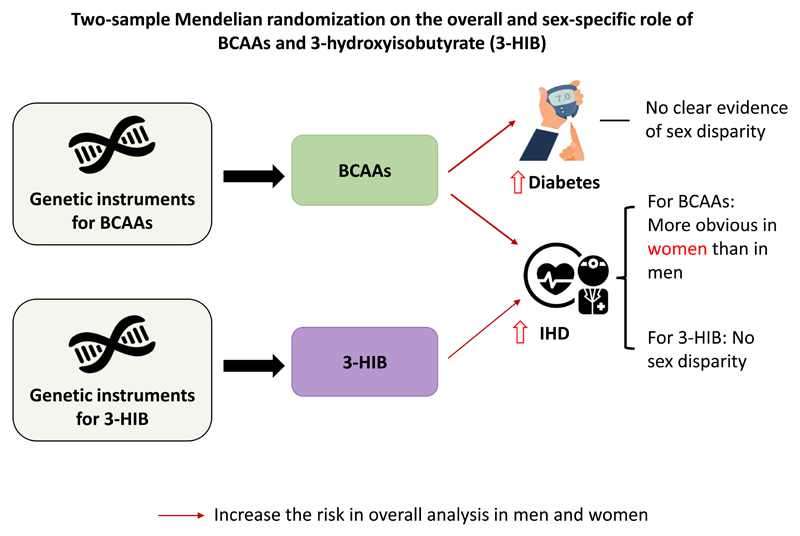
Graphical abstract

## Introduction

Ischemic heart disease (IHD) is a leading cause of death globally. As such, identifying more effective intervention targets, especially dietary interventions applicable in daily life, would be valuable for primary prevention and primary care. Branch-chain amino acids (BCAAs), including isoleucine, leucine, and valine, are building blocks for all life forms, and essential amino acids for humans.^1^ BCAAs have been studied for decades as agents related to ageing.^2^ BCAAs play an important role in protein synthesis and stimulation of cell signalling via activation of mammalian target of rapamycin (mTOR), with relevance to the balance of cell growth and autophagy.^3^ In contrast to the potential health promoting effects in the condition of negative energy balance,^2^ mounting studies have shown higher BCAAs may be associated with a higher risk of type 2 diabetes.^4^ Diabetes is an established risk factor for IHD, so it has been hypothesized that BCAAs may be associated with IHD. Observationally, BCAAs were associated with a higher risk of IHD.^5^ However, whether BCAAs are a biomarker or a causal factor for diabetes and IHD has not been clarified, as observational studies may be open to residual confounding and reverse causality. Moreover, BCAAs may affect the synthesis and/or metabolism of sex hormones. For example, *in vitro* experiments show that BCAAs may modulate estrogen biosynthesis,^6^ raising the possibility of an association with sex disparity, which has not been examined previously.

In addition to BCAAs, the valine metabolite, 3-hydroxyisobutyrate (3-HIB), also plays an important role in metabolism, but its role has been under-studied.^7^ In contrast to CoA-bound catabolites from leucine and isoleucine degradation, 3-HIB, the valine metabolite, is the only intermediate metabolite of BCAAs that is separated from its covalent attachment to CoA; consequently, it is the only such metabolite that can easily leave the mitochondrial matrix and enter the extracellular fluid.^1^ Observationally, 3-HIB is linked to diabetes,^8, 9^ whilst we have not identified previous studies specifically examining its association with IHD.

BCAAs are abundant in animal products, especially in red meat. BCAAs supplements are also widely used by athletes to increase muscle mass and enhance performance. Due to the unclear long-term health effects, current dietary guidelines have not provided clear recommendations of BCAAs intake.^10^ In the situation where evidence from large randomized controlled trials is not available, Mendelian randomization (MR) provides an approach to obtain unconfounded estimates in an observational setting, using naturally occurring genetic variants as instruments.^11^ As the genotypes are randomly allocated at conception, MR can minimize confounding from socioeconomic position and health status.^11^ Using this study design, previous studies suggest that genetically predicted higher BCAAs might be associated with higher risk of IHD^12^ and diabetes,^13^ however, previous studies used genetic instruments derived from relatively small GWAS of metabolites^13, 14^ (n=24,925 and 16,596 respectively),and have not examined the sex-specific associations. To our knowledge, there is no MR study on the role of 3-HIB in IHD and diabetes. With the emerging larger genome-wide association studies (GWAS) available, we examined the overall and sex-specific associations of genetically predicted BCAAs and 3-HIB with the risk of IHD and diabetes.

## Materials and Methods

### Study design

We conducted a two-sample MR study based on well-established large cohorts and consortia (Supplemental Table 1). Specifically, we applied genetic proxies for total BCAAs and 3-HIB to genome-wide association studies (GWAS) of IHD and diabetes. Given the potential sex differences, we also conducted sex-specific analysis. To be comprehensive, we also examined their role in cardiovascular disease (CVD) risk factors including blood glucose, lipids (triglycerides and low-density lipoprotein cholesterol (LDL-c)), body mass index (BMI) and blood pressure. For ease of comparison, we also conducted a conventional observational study in the UK Biobank. The data sources of all outcomes are shown in Supplemental Table 1.

### BCAAs and 3-HIB measurement and their genetic instruments

Blood BCAAs and 3-HIB were measured using a high-throughput NMR-based metabolic biomarker profiling platform developed by Nightingale Health Ltd. BCAAs and 3-HIB concentrations are correlated (correlation coefficient -0.036, p value 1.13×10^-28^). Genetic predictors for BCAAs and 3-HIB were obtained based on summary statistics of a large GWAS of metabolomics, including BCAAs and 3-HIB, in the UK Biobank based on 121,577 samples which were randomly selected and passed the quality control; all the selected samples were from people of European ancestry. In the GWAS, all metabolites were standardized prior to the analyses. Specifically, we selected single nucleotide polymorphisms (SNPs) associated with total BCAAs or 3-HIB at genome-wide significance (5x10^-8^) and with a linkage disequilibrium (LD) threshold of r^2^<0.001. To check the validity of these selected SNPs, we calculated the F-statistic, using a well-established formula.^15^ A cut-off of 10 is used as a “rule of thumb” to distinguish between strong and weak instruments.^16^ The selected SNPs were shown in Supplemental Tables 2 and 3. To check the potential pleiotropy, we also assessed the association of these selected SNPs with potential confounders in the association of BCAAs with IHD or diabetes, including Townsend index, education, smoking, alcohol drinking, and processed meat intake in the UK Biobank, and excluded SNP(s) associated with any of these factors at genome-wide significance in sensitivity analysis.

### Genetic associations with IHD and diabetes

In the overall analysis, summary genetic associations with IHD were obtained from a GWAS meta-analysis in CARDIoGRAMplusC4D (122,733 cases, 424,528 controls, including UK Biobank), mainly in people of European ancestry.^17^ Cases were defined using ICD code I21-I25 and the Office of Population Censuses and Surveys Classification of Interventions and Procedures, version 4 (OPCS-4) codes: K40-K46, K49, K50 and K75 which includes therapeutic operations on coronary artery; self-reported coronary heart disease was also used in the definition. Genetics associations with IHD were also taken from the FinnGen (31,640 cases, 187,152 controls), which used a similar definition. To improve power, the genetic associations with IHD from the two GWAS were meta-analyzed. Genetic associations with type 2 diabetes were obtained from DIAGRAM (74,124 cases and 824,006 controls, including UK Biobank)^18^ and FinnGen (35,607 cases and 183,185 controls) (Supplemental Table 1). Cases were defined based on fasting glucose or glycated haemoglobin levels, hospital discharge diagnosis, use of diabetes medication or self-report. Similarly, the genetic associations with diabetes were meta-analyzed.

In sex-specific analysis, we used individual-level data from UK Biobank for IHD, and sex-specific summary statistics from a GWAS meta-analysis in DIAGRAM (including UK Biobank) for diabetes. UK Biobank is a large, ongoing, prospective cohort study, with currently a median follow up time of 11.1 years.^19^ It recruited 502,713 people (intended to be aged 40-69 years, mean age 56.5 years, 45.6% men) from 2006 to 2010 in England, Scotland and Wales, 94% of self-reported European ancestry. Genotyping was assessed using two similar arrays, i.e., the UK BiLEVE array and UK Biobank Axiom array. To control for population stratification, the participants were restricted to those with white British ancestry. For quality control, participants were excluded if they fulfilled the following criteria: 1) have excess relatedness (more than 10 putative third-degree relatives); 2) have inconsistent information about sex based on genotyping and self-report; 3) have sex-chromosomes not XX or XY; 4) have poor-quality genotyping based on heterozygosity and missing rates; or 5) have withdrawn from UK Biobank. IHD and diabetes events were obtained from record linkage to hospitalization and death records, as well as a nurse-led interview at recruitment (i.e., prevalent cases), as previously.^20^ After quality control, 47,413 cases of IHD (31,127 in men, 16,286 in women) have been identified. To obtain the sex-specific associations with IHD, logistic regression controlling for age, assay array and 20 principal components was applied. In the sex-specific analysis for diabetes, the sex-specific associations with diabetes were based on GWAS meta-analysis including 41,846 cases and 383,767 controls in men, and 30,053 cases and 434,336 controls in women.^18^

### Genetic associations with CVD risk factors

In the overall analysis, genetic associations with fasting glucose were obtained from the Meta-Analyses of Glucose and Insulin-related traits Consortium (MAGIC) (n=200,622).^21^ Genetic associations with triglycerides and LDL-c were derived from the Global Lipids Genetics Consortium (GLGC) in participants of European ancestry, without participants of UK Biobank; the sample size is up to 0.86 and 0.84 million for triglycerides and LDL-c, respectively.^22^ Genetic associations with blood pressure obtained from the GWAS meta-analysis of UK Biobank with the International Consortium of Blood Pressure (ICBP) (n=757,601).^23^ Genetic associations with BMI were obtained from the UK Biobank (n=461,460).^24^ In the sex-specific analyses, sex-specific genetic associations with triglycerides and LDL-c were obtained from the GWAS meta-analysis in GLGC (including UK Biobank).^22^ The sex-specific associations with other outcomes were obtained from summary statistics in UK Biobank, provided by Neale Lab (http://www.nealelab.is/uk-biobank).

### Statistical analysis

After discarding palindromic SNPs (shown in Supplemental Tables 2 and 3), we calculated the Wald estimate (genetic association with IHD and diabetes divided by the genetic association with BCAAs or 3-HIB) for each SNP, and then combined using inverse variance weighting (IVW) with multiplicative random effects.^25^ In the analysis by sex, we used the same SNPs as in the overall analysis, but obtained sex-specific associations with exposures and outcomes. The analyses on CVD risk factors were conducted in a similar way. The MR estimates were presented as the odds ratio (OR) (for IHD and diabetes) or beta-coefficient (for continuous outcomes) per standard deviation (SD) increase in BCAAs or 3-HIB. To account for multiple comparisons, a Bonferroni correction was used, with corrected cut-off p-value of 0.05/2 (exposures)/8 (outcomes) =0.003). We considered associations with nominal significance, i.e., with p-value<0.05 but not reaching Bonferroni-corrected significance as suggestive associations.

To account for potential pleiotropy, we used several analytic methods robust to pleiotropy, including weighted median, weighted mode, Mendelian randomization pleiotropy residual sum and outlier (MR-PRESSO) and MR-Egger. The weighted median provides a consistent estimate of the causal effect even when up to 50% of the information is from genetic variants that are invalid instruments.^26^ The weighted mode is based on the assumption that a plurality of genetic variants are valid instruments, i.e., no larger subset of invalid instruments estimating the same causal parameter than the subset of valid instruments exists.^27^ MR-PRESSO was able to identify the genetic variant(s) differentially driving the associations, i.e., outliers,^28^ and provide corrected estimates removing the outliers. If so, corrected estimates after removing outliers, rather than from IVW, were presented. MR-Egger was able to assess whether genetic variants have pleiotropic effects on the outcome that differ on average from zero (directional pleiotropy), indicated by a non-zero intercept.^29^ To test and control the potential pleiotropy, we also examined the association of each genetic variant with sex hormone binding globulin (SHBG), the regulator of sex hormones, considering that SHBG may regulate BCAA metabolism^30^ and SHBG has been shown to be related to lower risk of diabetes and IHD in previous MR studies.^31, 32^ 3 SNPs for BCAAs (Supplemental Table 2) and 6 SNPs for 3-HIB (Supplemental Table 3) were related to SHBG. To understand whether the association was horizontal or vertical pleiotropy, we did a bi-directional MR on BCAAs or 3-HIB and SHBG. As shown in Supplemental Table 4, for BCAAs and SHBG, both directions are possible (i.e., both vertical and horizontal pleiotropy are possible), whilst 3-HIB affects SHBG rather than vice versa (i.e., vertical pleiotropy which does not violate MR assumption). As such, for BCAAs, we also conducted sensitivity analysis excluding the three SNPs related to SHBG. For the associations reaching Bonferroni-corrected significance or nominal significance, we also performed Steiger directionality test which enables inference of the causal direction.^33^

Power calculation was conducted based on the approximation that the sample size for a MR study is the sample size for exposure on outcome divided by the r^2^ for genetic proxies on exposure.^34, 35^ Sex difference was tested using heterogeneity test using “meta” package.

### Conventional observational study

For ease of comparison with MR, we also conducted logistic regression to assess the associations of BCAAs and 3-HIB with IHD and diabetes risk. The analyses were conducted in the participants with measurements of metabolites and outcomes, and the same exclusion criteria as in the MR study. In model 1, we controlled for age, sex, smoking, alcohol drinking, education, physical activity, Townsend index, processed meat intake, use of medication and baseline diseases. BMI is generally considered as a mediator which links the association of BCAAs with diabetes, however, as we cannot exclude the possibility of BMI being a confounder, in model 2 we additionally adjusted for BMI.

All statistical analyses were conducted using the “TwoSampleMR”, “MendelianRandomization”, “MRPRESSO” and “meta” packages in R (version 4.0.1, R Foundation for Statistical Computing, Vienna, Austria).

## Results

### Overall and sex-specific associations for BCAAs using MR

**Figure 1:**
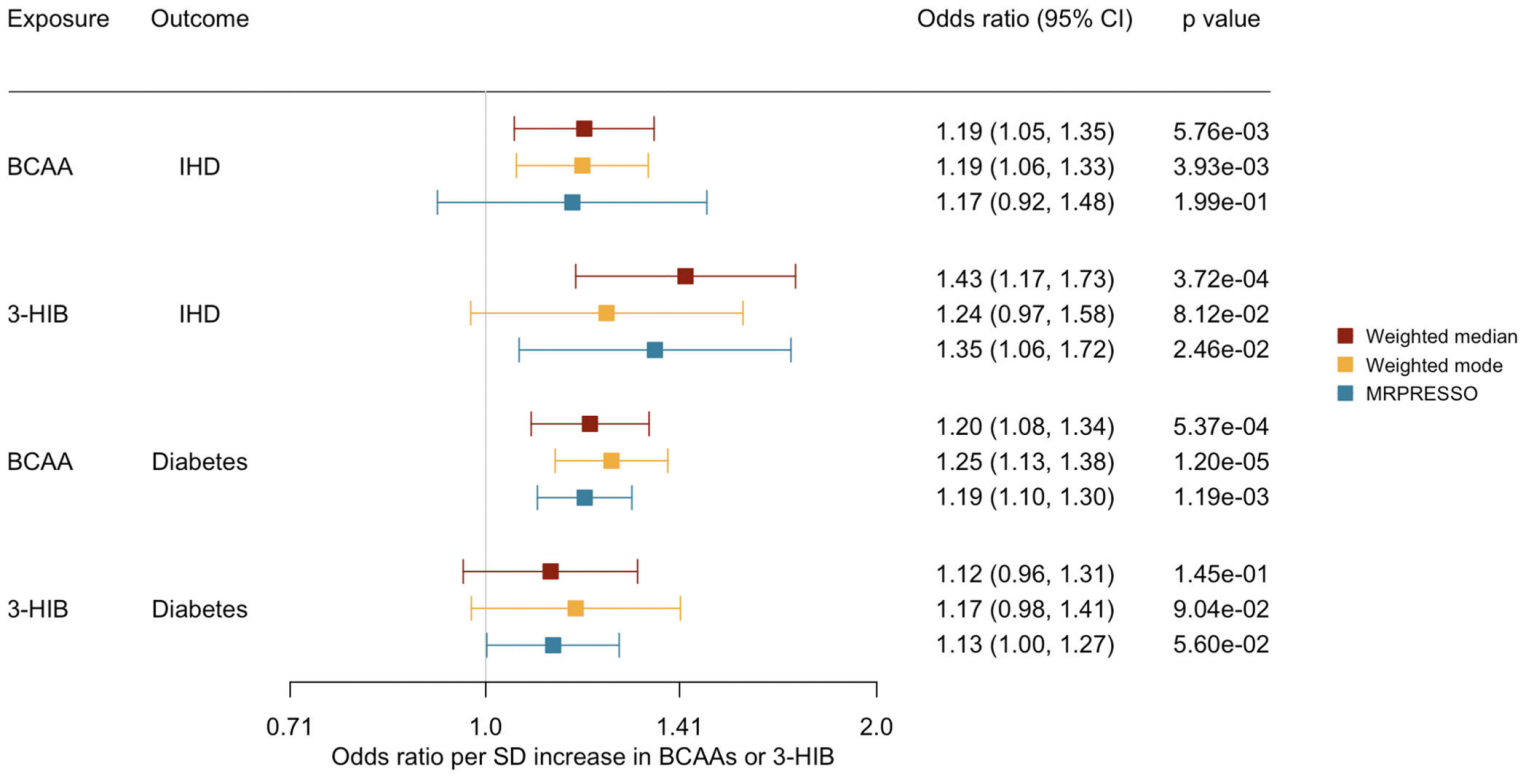
Association of genetically predicted BCAAs and 3-HIB with ischemic heart disease and diabetes using different analytic methods. When MR-PRESSO detected outliers, inverse variance weighting was not used, instead estimates from methods more robust to pleiotropy, including weighted median, weighed mode and MR-PRESSO, were shown.

17 genetic variants were identified for BCAAs (Supplemental Table 2) and 3-HIB (Supplemental Table 3). After excluding palindromic SNPs and SNPs not available in the outcome datasets (shown in Supplemental Table 2), 15 and 16 SNPs were used in the overall analysis for IHD and diabetes respectively, and all 17 SNPs were used in the sex-specific analysis. These SNPs were not associated with potential confounders, except for rs1260326,which was associated with alcohol consumption (Supplemental Table 5), so we excluded this SNP in sensitivity analysis. Genetically predicted higher BCAAs were associated with higher risk of IHD (with nominal significance) and diabetes in the overall analysis (Figure 1), with an OR of 1.19 (95% confidence interval (CI) 1.05 to 1.35 per SD increase in BCAAs) for IHD and OR of 1.20 (95% CI 1.08 to 1.34) for diabetes using weighted median, with similar estimates from weighted mode (Figure 1). The associations were also consistent in different data sources; for IHD, OR of 1.21, 95% CI 1.05 to 1.40 in CARDIoGRAMplusC4D and 1.18, 95% CI 0.97 to 1.43 in FinnGen, I^2^=0.0%, for diabetes, OR of 1.15, 95% CI 1.02 to 1.31 in DIAGRAM and 1.35, 95% CI 1.14 to 1.58 in FinnGen using weighted median, I^2^=54.3%. As MR-PRESSO detected outliers (Supplemental Table 6), which was also shown in scatter plots (Supplemental Figure 1), estimates from IVW were not used. The overall association with IHD included the null using MR-PRESSO (Figure 1), however, the directions of associations are consistent. MR-Egger gave wider confidence intervals than other methods. MR-Egger did not indicate directional pleiotropy (all intercept p values>0.05) (Supplemental Table 7). The causal direction was supported by Steiger test. The findings remained after excluding the potentially pleiotropic SNP rs1260326 (Supplemental Figure 2). The associations also remained after excluding the three SNPs related to SHBG (Supplemental Table 8).

**Figure 2:**
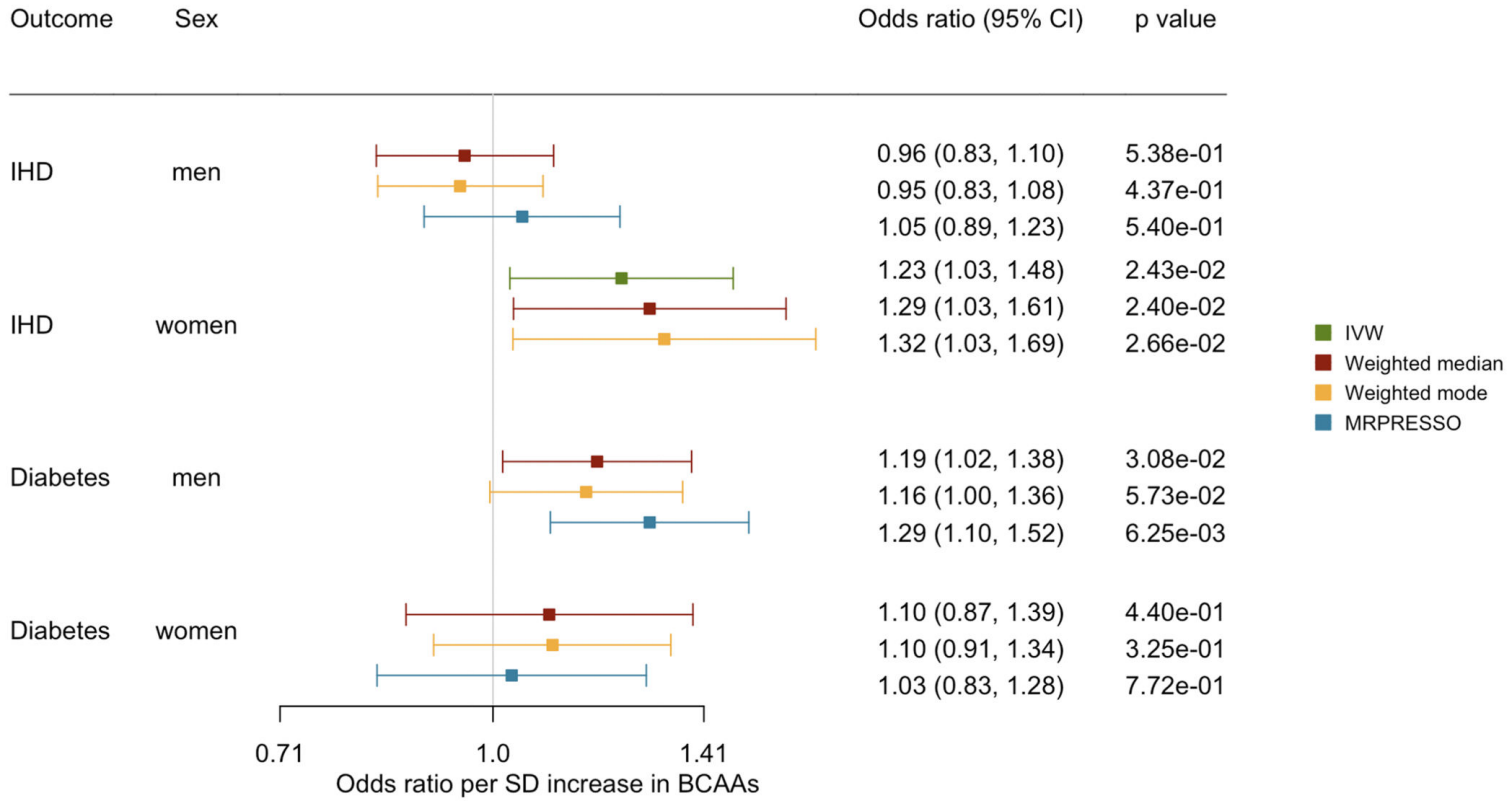
Sex-specific association of genetically predicted BCAAs with IHD and diabetes using different analytic methods

In the sex-specific analysis, genetically predicted BCAAs were associated with a higher risk of IHD in women but not in men (p value for sex difference=0.03) (Figure 2). Genetically predicted BCAAs were associated with a higher risk of diabetes in men but not in women, but the sex difference did not reach statistical significance (p value for sex difference=0.15) (Figure 2). The associations were consistent using different analytic methods (Figure 2) and after removing rs1260326 (Supplemental Figure 3) or SNPs related to SHBG (Supplemental Table 8).

Regarding CVD risk factors, the overall and sex-specific associations were shown in Supplemental Figures 4 and 5. Genetically predicted BCAAs were nominally related to higher glucose in men but not in women, and nominally associated with higher triglycerides using MR-PRESSO overall and in men. We found suggestive associations of BCAAs with higher systolic blood pressure (SBP) overall (robust to different methods) and in men (using MR-PRESSO), as well as with higher diastolic blood pressure (DBP) in women (using weighted median and weight mode). The outliers detected by MR-PRESSO were shown in Supplemental Table 9. Genetically predicted BCAAs were related to higher BMI overall, in men (only when using weighted median and weight mode) and in women (robust to different methods). MR-Egger did not detect directional pleiotropy (Supplemental Table 10). Steiger test supported the causal direction for all associations except for the overall association with SBP. We found no sex difference for these associations.

### Overall and sex-specific associations for 3-HIB using MR

17 SNPs were identified for 3-HIB, with an average F-statistic of 62.9 (Supplemental Table 3); only 1 SNP was shared with BCAAs. After excluding palindromic SNPs and SNPs not available in the outcome datasets (shown in Supplemental Table 3), 14 and 16 SNPs were used in the overall analysis for IHD and diabetes respectively, and all 17 SNPs were used in the sex-specific analysis. All genetic variants were not associated with the potential confounders (Supplemental Table 11). In the overall analysis, genetically predicted higher 3-HIB was associated with a higher risk of IHD using weighted median (Figure 1). The associations were consistent in different data sources, with OR of 1.38, 95% CI 1.11 to 1.72 in CARDIoGRAMplusC4D and 1.40, 95% CI 1.07to 1.84 in FinnGen, I^2^=0.0%. The associations using weighted mode and MR-PRESSO had wider confidence intervals and did not reach Bonferroni-corrected significance, but the confidence intervals overlap with that in weighted median. MR-Egger did not indicate directional pleiotropy (Supplemental Table 12). The causal direction was supported by Steiger test. Genetically predicted 3 -HIB was not associated with diabetes (Figure 1).

**Figure 3:**
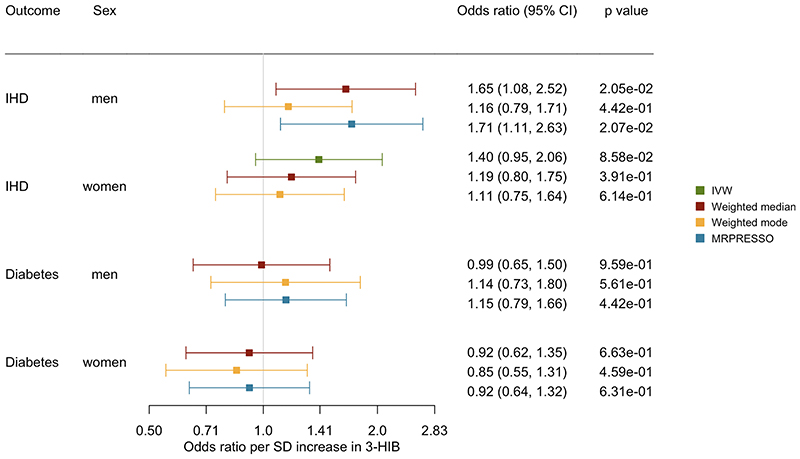
Sex-specific association of genetically predicted 3-HIB with IHD and diabetes using different analytic methods

In the sex-specific analysis, genetically predicted 3-HIB was associated with a higher risk of IHD in men but not in women (Figure 3). However, the sex difference did not reach statistical difference (p value for sex difference=0.26). Genetically predicted 3-HIB was not related to diabetes in either men or women (Figure 3), with consistent findings using different analytic methods.

Regarding CVD risk factors, the overall and sex-specific associations were shown in Supplemental Figures 6 and 7. Genetically predicted 3-HIB was associated with lower triglycerides and LDL-c overall and in women (only when using weighted mode), but the causal direction was not supported by Steiger test except for LDL-c overall analysis. The associations did not differ by sex. The outliers detected by MR-PRESSO were shown in Supplemental Table 13. MR-Egger did not indicate directional pleiotropy except for the overall association with SBP and the association with SBP and DBP in men; corrected MR-Egger estimates suggested a positive association of genetically predicted 3-HIB with SBP overall and in men (Supplemental Table 14). Genetically predicted 3-HIB was not associated with other CVD risk factors.

Power calculation results for BCAAs and 3-HIB were shown in Supplemental Table 15. For both BCAAs and 3-HIB, power was slightly higher for analyses in men than in women.

### Conventional observational study

Using conventional observational analysis (Table 1), we found BCAAs were associated with higher risk of IHD and diabetes overall, in men and in women. The associations showed no difference by sex. 3-HIB was associated with higher risk of IHD overall, and possibly in men and in women, but not with diabetes. The estimates were similar with and without controlling for BMI.

## Discussion

Using MR to minimize confounding, this study, together with previous evidence, support an unfavorable association of BCAAs with IHD and diabetes. In addition, this study adds to the limited evidence on their sex-specific associations with IHD and diabetes, by showing that BCAAs may have a stronger association with IHD in women. To our knowledge, this is the first MR study to investigate the overall and sex-specific associations of 3-HIB with IHD and diabetes. Genetically predicted 3-HIB was associated with a higher risk of IHD, with no sex disparity (Figure 4).

Our findings on the overall associations of BCAAs with IHD and diabetes using MR are consistent with published observational studies,^4, 5^ and our conventional observational study in the UK Biobank. The positive association of genetically predicted BCAAs with IHD was consistent with an MR study^12^ based on 17 different SNPs (with F-statistics above 10) derived from a smaller GWAS of metabolites including BCAAs (n=24,925);^14^ the association with diabetes was also consistent with an MR study based on limited number of genetic instruments (5 SNPs for isoleucine, 1 SNP for leucine and 1 SNP for valine, with F-statistics above 10), derived from a smaller GWAS of BCAAs (n=16,596).^13^ The evidence regarding the sex-specific associations is limited. Compared with BCAAs, 3-HIB has been less examined.^7^ Our study adds to the limited evidence and shows that it may also be a target for IHD.

Several mechanisms might underlie the role of BCAAs and 3-HIB in IHD and diabetes. In animal experiments, BCAAs transcriptionally upregulated PPAR-α expression, thereby exacerbated lipid peroxidation toxicity and cardiac ischemia/reperfusion vulnerability.^36^ BCAAs also regulate the mechanistic target of rapamycin (mTOR) pathway,^1^ which is crucial for cardiometabolism.^37^ BMI may also mediate the association of BCAAs with diabetes, however, MR analysis using different analytic methods did not confirm BCAAs affect BMI (Supplemental Table 16). If BMI is a mediator, then the association of BCAAs with diabetes may vary with BMI. However, in the observational analysis we did not detect the interaction (p value for interaction term is 0.42). The MR models with and without controlling for BMI also gave consistent findings. 3-HIB was secreted from the muscle boosted by catabolic flux of BCAAs. It may activate the endothelial fatty acid transport, stimulate muscle fatty acid uptake in vivo and promote lipid accumulation in muscle in mice,^38^ possibly leading to increased risk of cardiometabolic diseases. Interestingly, we found a potential sex disparity in the associations of BCAAs with IHD. Genetically predicted BCAAs were related to diabetes in men but not in women, possibly due to the lower power in women than in men, with no statistical difference. Consistently, we found no statistical difference in the sex-specific associations with blood glucose. It is possible that the mediators involved in the pathway from BCAAs to IHD and/or diabetes may have sex-specific roles. For example, BCAAs are well known to be prime activators of mTOR,^7, 39^ which may be involved in the pathway. mTOR regulates sex hormones and reproduction in men and possibly in women,^40^ and may exert sex-specific effects.^41^ However, these pathways remain to be tested in mechanistic trials.

Despite the novelty, these findings need to be interpreted cautiously. First, MR studies assess lifelong associations for endogenous exposures, rather than the effect of an intervention. As such, these associations may not be interpreted as the short-term effect of BCAAs or 3-HIB supplementation. These findings in Europeans might also not be applicable to other ancestries, such as Asians and Africans. Moreover, MR estimates, although less confounded, are less precise than conventional observational studies, because the genetic variants only explain a small proportion of the variance in exposure.^34^ The statistical testing for the sex disparity in the association of BCAAs with diabetes was at a marginal significance; we cannot exclude the possibility that it is due to a lack of power to detect the sex difference. Men under 65 years old have lower number of competing risk factors and comorbidities than older men, so it is possible that the effects of BCAAs on IHD or diabetes in men vary with age. However, the analysis had limited power due to the lower number of cases after stratification by age at recruitment in men. Similarly, for the null associations in sex-specific analysis, we cannot exclude the possibility of an association with a lower effect size than we can detect. Replication in a larger sample would be worthwhile. Thirdly, in sex-specific analyses we did not derive sex-specific genetic instruments, as the currently available GWAS of exposures were conducted in overall populations. However, we used sex-specific associations with exposures in the analysis, and we did not detect sex difference in the gene - exposure associations. Fourthly, the sample of exposures and outcomes partly overlap, for example, the data sources for IHD and BCAAs both include UK Biobank, with ~15% overlapping. However, a recent simulation study supported the validity of overlapping samples in large cohorts, such as UK Biobank.^42^ In addition, MR requires stringent assumptions, i.e., the genetic variants are associated with the exposure, no confounders of the associations of the genetic variants with the outcomes exist, and the genetic variants are not associated with the outcomes other than via affecting the relevant exposure (no pleiotropy).^11^ To satisfy these assumptions, we only selected SNPs strongly associated with BCAAs or 3-HIB. The leading SNP for BCAAs, rs10018448 close to the gene *PPM1K,* was also related to the ratio of valine to 3-HIB (p=1.2×10^-8^) and functionally relevant to the catabolism of BCAAs. PPM1K encodes the mitochondrial phosphatase that activates the branched-chain alpha-ketoacid dehydrogenase (BCKD) complex, which is responsible for the rate-limiting step of BCAA catabolism.^13^ Population stratification might be a confounder for MR estimates. However, the genetic associations with BCAAs and 3 -HIB as well as with IHD and diabetes are all from studies in people of European descent, with genomic control. We also used different analytic methods robust to pleiotropy, which gave a similar interpretation. Our findings on the overall effect of BCAAs are also consistent with two previous MR studies using different genetic instruments,^12, 13^ the consistency also adds to the validity of the findings. Finally, the relatively small effect size might not be of clinical significance. However, relatively small effects of causal factors may still be an important determinant of population health,^43^ particularly for foods consumed in daily life.

From the perspective of public health and clinical practice, our findings suggest BCAAs and 3-HIB might be a target of intervention for IHD. Diet rich in BCAAs and 3-HIB, such as red meat, and BCAAs supplements are not recommended for people with or at high risk of IHD and/or diabetes. Meanwhile, given the sex-specific role of BCAAs, it is worthwhile to consider sex disparity in dietary recommendations, and to explore the underlying pathways.

## Supplementary Material

Supplementary material

## Figures and Tables

**Table 1:** Conventional observational study on the overall and sex-specific associations of BCAAs and 3-HIB with IHD and diabetes in the UK Biobank

## Data Availability

Data described in the manuscript will be available upon request and approval by the UK Biobank (https://www.ukbiobank.ac.uk/enable-your-research/apply-for-access). Other data used in the manuscript are publicly available.
