# Supplementary material for: Using genetics to examine the overall and sex-specific associations of branch-chain amino acids and the valine metabolite, 3-hydroxyisobutyrate, with ischemic heart disease and diabetes: a two-sample Mendelian randomization study
Source: Atherosclerosis. Author manuscript; Available in PMC 2023 Sep 8. (PMC7615055; doi:10.1016/j.atherosclerosis.2023.117246)

Supplemental Table 1. Data sources used in this study

| Outcome | Source | Ncase | Ncontrol |
| --- | --- | --- | --- |
| *Overall* |  |  |  |
| IHD | CARDIoGRAMplusC4D | 122,733 | 424,528 |
|  | FinnGen | 31,640 | 187,152 |
| Diabetes | DIAGRAM | 74,124 | 824,006 |
|  | FinnGen | 35,607 | 183,185 |
|  |  | Sample size |  |
| Glucose | MAGIC | 200,622 |  |
| LDL-c and | GLGC | Up to 0.84 million | |
| Triglycerides | GLGC | Up to 0.86 million | |
| Blood pressure | UK Biobank and ICBP meta-analysis | 757,601 |  |
| Body mass index | UK Biobank | 461,460 |  |
|  |  |  |  |
| *Sex-specific* |  | Ncase | Ncontrol |
| IHD in men | UK Biobank (individual data) | 31,127 | 148,777 |
| IHD in women | UK Biobank (individual data) | 16,286 | 195,774 |
| Diabetes in men | DIAGRAM | 41,846 | 383,767 |
| Diabetes in women | DIAGRAM | 30,053 | 434,336 |
|  |  | Sample size |  |
| Glucose, blood pressure and BMI | UK Biobank | 194,174 in men | 167,020 in women |
| LDL-c and triglycerides | GLGC | Up to 686,787 in men | Up to 570,542 in women |

Supplemental Table 2. Genetic predictors for BCAAs

| SNP | Chr | Pos | Gene | EA | OA | Beta | SE | P value | F-statistic |
| --- | --- | --- | --- | --- | --- | --- | --- | --- | --- |
| rs2943652 | 2 | 227108446 | *NYAP2, MIR5702* | T | C | 0.02 | 0.004 | 2.20E-08 | 31.3 |
| rs1128249 | 2 | 165528624 | *COBLL1* | T | G | -0.03 | 0.004 | 2.00E-17 | 72.2 |
| rs1260326 | 2 | 27730940 | *GCKR* | C | T | -0.05 | 0.004 | 2.70E-36 | 158.3 |
| rs72538440 | 2 | 65225088 | *LINC02245,*  *SLC1A4* | G | GC | -0.05 | 0.004 | 4.60E-30 | 129.8 |
| rs34894639 | 3 | 135798658 | *PPP2R3A* | T | C | -0.03 | 0.005 | 3.50E-09 | 34.9 |
| rs10018448 | 4 | 89231404 | *Close to PPM1K* | G | A | 0.10 | 0.004 | 7.29E-128 | 578.7 |
| rs7656569^*^ | 4 | 88245609 | *ABCG2-PPM1K* | A | C | 0.06 | 0.005 | 7.40E-36 | 156.3 |
| rs2977929 | 8 | 76454025 | *HNF4G* | T | C | 0.03 | 0.005 | 1.10E-08 | 32.7 |
| rs17096421 | 10 | 88820592 | *GLUD1* | T | A | 0.05 | 0.009 | 9.70E-10 | 37.4 |
| rs2638315 | 12 | 56865056 | *SPRYD4, GLS2* | C | G | 0.04 | 0.005 | 1.80E-17 | 72.4 |
| rs36181536 | 12 | 122528263 | *MLXIP* | C | T | -0.03 | 0.004 | 7.00E-10 | 38.0 |
| rs6499561 | 16 | 72149061 | *PMFBP1* | G | A | 0.02 | 0.004 | 1.30E-08 | 32.3 |
| rs77724138 | 16 | 70207520 | *CLEC18C* | C | G | -0.09 | 0.006 | 2.90E-51 | 226.9 |
| rs117643180 | 17 | 7185779 | *SLC2A4* | A | C | -0.15 | 0.013 | 3.80E-32 | 139.3 |
| rs12974412 | 19 | 14151809 | *IL27RA* | G | A | -0.03 | 0.005 | 1.80E-08 | 31.7 |
| rs4801776 | 19 | 49304215 | *BCAT2* | T | C | -0.04 | 0.004 | 1.30E-18 | 77.6 |
| rs5747934^*^ | 22 | 18927769 | *PRODH* | T | C | -0.06 | 0.010 | 1.20E-09 | 37.0 |

^*^rs5747934 was not available in the outcome; rs7656569 was not used for ischemic heart disease due to incompatible alleles.

rs2943652, rs34894639 and rs1128249 were also related to SHBG.

Supplemental Table 3. Genetic predictors for 3-HIB

| SNP | Chr | Pos | Gene | EA | OA | Beta | SE | P value | F-statistic |
| --- | --- | --- | --- | --- | --- | --- | --- | --- | --- |
| rs10127775^*^ | 1 | 230160042 | *GALNT2* | T | A | -0.03 | 0.004 | 8.30E-13 | 51.2 |
| rs585188 | 5 | 41702897 | *PLCXD3, OXCT1* | A | G | -0.05 | 0.005 | 2.70E-22 | 94.3 |
| rs9987289 | 8 | 9183358 | *RNU6-1151P, RNU6-526P* | G | A | 0.07 | 0.007 | 7.30E-20 | 83.2 |
| rs2645433^*^ | 8 | 11800412 | *FDFT1* | C | G | -0.03 | 0.004 | 8.80E-11 | 42.1 |
| rs4149307 | 9 | 107589744 | *ABCA1* | T | C | 0.03 | 0.006 | 9.30E-10 | 37.5 |
| rs2575876 | 9 | 107665739 | *ABCA1* | A | G | -0.03 | 0.005 | 2.50E-11 | 44.5 |
| rs2419604 | 10 | 113944271 | *GPAM* | G | A | 0.04 | 0.005 | 6.90E-19 | 78.8 |
| rs964184 | 11 | 116648917 | *ZPR1* | C | G | -0.05 | 0.006 | 1.20E-15 | 64.0 |
| rs1169297^*^ | 12 | 120990652 | *HNF1A* | A | G | -0.02 | 0.004 | 2.70E-08 | 30.9 |
| rs12314700 | 12 | 122490043 | *BCL7A* | T | A | -0.04 | 0.005 | 4.80E-11 | 43.3 |
| rs72702354 | 14 | 94643207 | *PPP4R4* | G | A | -0.07 | 0.010 | 2.80E-11 | 44.3 |
| rs28929474 | 14 | 94844947 | *SERPINA1* | T | C | -0.16 | 0.015 | 7.50E-27 | 115.1 |
| rs473224 | 15 | 58737341 | *LIPC-AS1, LIPC, ALDH1A2* | G | T | 0.03 | 0.006 | 8.80E-09 | 33.1 |
| rs35853021 | 15 | 58680643 | *ALDH1A2* | T | G | -0.03 | 0.004 | 1.10E-11 | 46.2 |
| rs9930957 | 16 | 72149923 | *PMFBP1* | T | C | 0.06 | 0.006 | 2.70E-29 | 126.3 |
| rs117643180 | 17 | 7185779 | *SLC2A4* | A | C | -0.08 | 0.013 | 8.20E-11 | 42.2 |
| rs429358 | 19 | 45411941 | *APOE* | C | T | 0.05 | 0.006 | 7.30E-22 | 92.3 |

^*^rs10127775 and rs2645433 were removed due to palindromic SNPs. rs1169297 was not available in the outcome. rs1169297, rs28929474, rs9987289, rs429358, rs2419604 and rs72702354 were related to SHBG.

Supplemental Table 4. Bi-directional association of BCAAs and SHBG, and 3-HIB and SHBG using bi-directional MR

| Exposure | Outcome | Beta | 95% CI | *p* |
| --- | --- | --- | --- | --- |
| BCAAs | SHBG | -0.03 | -0.05, -0.01 | 2.6E-4 |
| SHBG | BCAAs | -0.23 | -0.32, -0.15 | 5.0E-9 |
| 3-HIB | SHBG | 0.03 | 0.00, 0.05 | 0.05 |
| SHBG | 3-HIB | 0.05 | -0.03, 0.13 | 0.19 |

The estimates were from weighted median which is robust to pleiotropy.

Supplemental Table 5. Genetic associations of BCAAs with potential confounders

| Potential confounders | SNP | chr | EA | OA | Beta | SE | P value |
| --- | --- | --- | --- | --- | --- | --- | --- |
| Age completed full time education | rs10018448 | 4 | G | A | 0.000 | 0.002 | 9.13E-01 |
| Alcohol intake frequency | rs10018448 | 4 | G | A | 0.004 | 0.004 | 2.93E-01 |
| Current tobacco smoking | rs10018448 | 4 | G | A | -0.001 | 0.001 | 6.12E-01 |
| Processed meat intake | rs10018448 | 4 | G | A | -0.001 | 0.002 | 5.60E-01 |
| Townsend deprivation index at recruitment | rs10018448 | 4 | G | A | -0.003 | 0.002 | 1.87E-01 |
| Age completed full time education | rs1128249 | 2 | T | G | 0.000 | 0.002 | 8.96E-01 |
| Alcohol intake frequency | rs1128249 | 2 | T | G | -0.015 | 0.004 | 6.24E-05 |
| Current tobacco smoking | rs1128249 | 2 | T | G | -0.002 | 0.001 | 2.09E-01 |
| Processed meat intake | rs1128249 | 2 | T | G | -0.004 | 0.002 | 9.10E-02 |
| Townsend deprivation index at recruitment | rs1128249 | 2 | T | G | 0.000 | 0.002 | 9.85E-01 |
| Age completed full time education | rs117643180 | 17 | A | C | -0.015 | 0.008 | 4.81E-02 |
| Alcohol intake frequency | rs117643180 | 17 | A | C | 0.008 | 0.011 | 4.62E-01 |
| Current tobacco smoking | rs117643180 | 17 | A | C | 0.003 | 0.004 | 4.94E-01 |
| Processed meat intake | rs117643180 | 17 | A | C | 0.019 | 0.007 | 5.30E-03 |
| Townsend deprivation index at recruitment | rs117643180 | 17 | A | C | -0.002 | 0.007 | 7.92E-01 |
| Age completed full time education | rs1260326 | 2 | C | T | 0.003 | 0.002 | 2.64E-01 |
| Alcohol intake frequency | rs1260326 | 2 | C | T | -0.048 | 0.004 | 7.60E-40 |
| Current tobacco smoking | rs1260326 | 2 | C | T | -0.002 | 0.001 | 1.10E-01 |
| Processed meat intake | rs1260326 | 2 | C | T | -0.009 | 0.002 | 1.10E-05 |
| Townsend deprivation index at recruitment | rs1260326 | 2 | C | T | 0.003 | 0.002 | 2.68E-01 |
| Age completed full time education | rs12974412 | 19 | G | A | 0.003 | 0.003 | 2.75E-01 |
| Alcohol intake frequency | rs12974412 | 19 | G | A | -0.005 | 0.004 | 2.78E-01 |
| Current tobacco smoking | rs12974412 | 19 | G | A | -0.001 | 0.002 | 3.99E-01 |
| Processed meat intake | rs12974412 | 19 | G | A | -0.001 | 0.003 | 7.40E-01 |
| Townsend deprivation index at recruitment | rs12974412 | 19 | G | A | -0.002 | 0.003 | 5.46E-01 |
| Age completed full time education | rs17096421 | 10 | T | A | 0.009 | 0.005 | 8.99E-02 |
| Alcohol intake frequency | rs17096421 | 10 | T | A | 0.004 | 0.008 | 6.27E-01 |
| Current tobacco smoking | rs17096421 | 10 | T | A | -0.001 | 0.003 | 6.82E-01 |
| Processed meat intake | rs17096421 | 10 | T | A | 0.000 | 0.005 | 9.90E-01 |
| Townsend deprivation index at recruitment | rs17096421 | 10 | T | A | -0.004 | 0.005 | 3.99E-01 |
| Age completed full time education | rs2638315 | 12 | C | G | -0.004 | 0.003 | 1.65E-01 |
| Alcohol intake frequency | rs2638315 | 12 | C | G | 0.008 | 0.005 | 7.17E-02 |
| Current tobacco smoking | rs2638315 | 12 | C | G | 0.001 | 0.002 | 5.90E-01 |
| Processed meat intake | rs2638315 | 12 | C | G | 0.010 | 0.003 | 2.70E-04 |
| Townsend deprivation index at recruitment | rs2638315 | 12 | C | G | 0.002 | 0.003 | 4.79E-01 |
| Age completed full time education | rs2943652 | 2 | T | C | 0.000 | 0.003 | 9.57E-01 |
| Alcohol intake frequency | rs2943652 | 2 | T | C | 0.011 | 0.004 | 2.45E-03 |
| Current tobacco smoking | rs2943652 | 2 | T | C | -0.002 | 0.001 | 1.49E-01 |
| Processed meat intake | rs2943652 | 2 | T | C | 0.005 | 0.002 | 1.20E-02 |
| Townsend deprivation index at recruitment | rs2943652 | 2 | T | C | 0.002 | 0.002 | 3.53E-01 |
| Age completed full time education | rs2977929 | 8 | T | C | 0.002 | 0.003 | 4.58E-01 |
| Alcohol intake frequency | rs2977929 | 8 | T | C | 0.015 | 0.004 | 7.00E-04 |
| Current tobacco smoking | rs2977929 | 8 | T | C | 0.000 | 0.002 | 9.35E-01 |
| Processed meat intake | rs2977929 | 8 | T | C | 0.005 | 0.003 | 6.60E-02 |
| Townsend deprivation index at recruitment | rs2977929 | 8 | T | C | 0.001 | 0.003 | 8.55E-01 |
| Age completed full time education | rs34894639 | 3 | T | C | 0.002 | 0.003 | 5.05E-01 |
| Alcohol intake frequency | rs34894639 | 3 | T | C | 0.008 | 0.004 | 5.93E-02 |
| Current tobacco smoking | rs34894639 | 3 | T | C | 0.001 | 0.002 | 5.57E-01 |
| Processed meat intake | rs34894639 | 3 | T | C | -0.006 | 0.002 | 1.50E-02 |
| Townsend deprivation index at recruitment | rs34894639 | 3 | T | C | 0.005 | 0.003 | 6.56E-02 |
| Age completed full time education | rs36181536 | 12 | C | T | 0.004 | 0.003 | 1.43E-01 |
| Alcohol intake frequency | rs36181536 | 12 | C | T | 0.002 | 0.004 | 5.71E-01 |
| Current tobacco smoking | rs36181536 | 12 | C | T | 0.001 | 0.001 | 6.67E-01 |
| Processed meat intake | rs36181536 | 12 | C | T | -0.003 | 0.002 | 1.60E-01 |
| Townsend deprivation index at recruitment | rs36181536 | 12 | C | T | 0.004 | 0.003 | 8.10E-02 |
| Age completed full time education | rs4801776 | 19 | T | C | -0.002 | 0.003 | 4.24E-01 |
| Alcohol intake frequency | rs4801776 | 19 | T | C | -0.012 | 0.004 | 1.90E-03 |
| Current tobacco smoking | rs4801776 | 19 | T | C | 0.000 | 0.001 | 8.05E-01 |
| Processed meat intake | rs4801776 | 19 | T | C | -0.008 | 0.002 | 4.30E-04 |
| Townsend deprivation index at recruitment | rs4801776 | 19 | T | C | 0.000 | 0.003 | 9.01E-01 |
| Age completed full time education | rs5747934 | 22 | T | C | -0.004 | 0.006 | 5.09E-01 |
| Alcohol intake frequency | rs5747934 | 22 | T | C | 0.005 | 0.009 | 5.91E-01 |
| Current tobacco smoking | rs5747934 | 22 | T | C | 0.001 | 0.003 | 7.69E-01 |
| Processed meat intake | rs5747934 | 22 | T | C | -0.011 | 0.005 | 3.80E-02 |
| Townsend deprivation index at recruitment | rs5747934 | 22 | T | C | 0.014 | 0.006 | 1.34E-02 |
| Age completed full time education | rs6499561 | 16 | G | A | -0.002 | 0.003 | 4.28E-01 |
| Alcohol intake frequency | rs6499561 | 16 | G | A | 0.004 | 0.004 | 3.48E-01 |
| Current tobacco smoking | rs6499561 | 16 | G | A | 0.004 | 0.001 | 3.47E-03 |
| Processed meat intake | rs6499561 | 16 | G | A | 0.003 | 0.002 | 1.40E-01 |
| Townsend deprivation index at recruitment | rs6499561 | 16 | G | A | 0.005 | 0.002 | 6.65E-02 |
| Age completed full time education | rs7656569 | 4 | A | C | -0.006 | 0.003 | 5.44E-02 |
| Alcohol intake frequency | rs7656569 | 4 | A | C | -0.001 | 0.005 | 8.98E-01 |
| Current tobacco smoking | rs7656569 | 4 | A | C | 0.000 | 0.002 | 7.78E-01 |
| Processed meat intake | rs7656569 | 4 | A | C | 0.000 | 0.003 | 9.20E-01 |
| Townsend deprivation index at recruitment | rs7656569 | 4 | A | C | 0.002 | 0.003 | 5.04E-01 |

Supplemental Table 6. Outliers detected by MR-PRESSO in the overall and sex-specific associations of BCAAs and 3-HIB with IHD and diabetes

| **Exposure** | **Outcome** | **Sex** | **Outlier SNPs** |
| --- | --- | --- | --- |
| BCAAs | IHD | Overall | rs10018448, rs1260326, rs34894639, rs6499561 |
| BCAAs | Diabetes | Overall | rs1128249, rs117643180, rs1260326, rs2943652, rs34894639 |
| 3-HIB | IHD | Overall | rs2419604, rs429358, rs473224, rs964184 |
| 3-HIB | Diabetes | Overall | rs117643180, rs2419604, rs28929474, rs35853021, rs429358, rs9987289 |
| BCAAs | IHD | men | rs10018448, rs1260326, rs2943652, rs34894639 |
| BCAAs | IHD | women | No outliers detected by MR-PRESSO |
| BCAAs | Diabetes | men | rs1128249, rs1260326, rs2943652 |
| BCAAs | Diabetes | women | rs1128249, rs1260326, rs2943652, rs34894639 |
| 3-HIB | IHD | men | rs1169297, rs35853021, rs429358 |
| 3-HIB | IHD | women | No outliers detected by MR-PRESSO |
| 3-HIB | Diabetes | men | rs429358 |
| 3-HIB | Diabetes | women | rs429358, rs964184, rs9987289 |

Supplemental Table 7. Overall and sex-specific associations of genetically predicted BCAAs with IHD and diabetes using MR-Egger

| Outcome | Sex | OR | 95% CI | *p* value | Intercept *p* value |
| --- | --- | --- | --- | --- | --- |
| IHD | Overall | 1.02 | 0.71, 1.48 | 0.90 | 0.16 |
| Diabetes | Overall | 0.87 | 0.50, 1.54 | 0.64 | 0.16 |
| IHD | men | 0.77 | 0.52, 1.14 | 0.20 | 0.03 |
| IHD | women | 1.09 | 0.72, 1.64 | 0.68 | 0.51 |
| Diabetes | men | 0.78 | 0.33, 1.86 | 0.58 | 0.25 |
| Diabetes | women | 0.67 | 0.25, 1.83 | 0.44 | 0.23 |

Supplemental Table 8. Overall and sex-specific association of genetically predicted BCAAs with IHD and diabetes using MR after excluding 3 SNPs related to SHBG^*^

| Outcome | Sex | Methods | OR | 95% CI | *p* |
| --- | --- | --- | --- | --- | --- |
| IHD | Overall | Weighed median | 1.17 | 1.04, 1.31 | 0.009 |
|  |  | Weighed mode | 1.18 | 1.04, 1.33 | 0.01 |
| Diabetes | Overall | Weighed median | 1.18 | 1.05, 1.32 | 0.005 |
|  |  | Weighed mode | 1.25 | 1.12, 1.38 | 2.9E-5 |
| IHD | Men | Weighed median | 0.95 | 0.82, 1.09 | 0.44 |
|  |  | Weighed mode | 0.94 | 0.82, 1.09 | 0.41 |
| IHD | Women | Weighed median | 1.26 | 1.00, 1.57 | 0.049 |
|  |  | Weighed mode | 1.32 | 1.01, 1.71 | 0.04 |
| Diabetes | Men | Weighed median | 1.25 | 1.04, 1.50 | 0.02 |
|  |  | Weighed mode | 1.26 | 1.05, 1.51 | 0.01 |
| Diabetes | Women | Weighed median | 0.96 | 0.75, 1.24 | 0.76 |
|  |  | Weighed mode | 1.07 | 0.81, 1.40 | 0.65 |

^*^The 3 SNPs refer to rs2943652, rs34894639 and rs1128249

Supplemental Table 9. Outliers detected in MR-PRESSO in the overall and sex-specific associations of BCAAs with CVD risk factors

| Outcome | sex | Outliers |
| --- | --- | --- |
| Glucose | overall | rs10018448,rs1128249,rs1260326,rs2638315,rs2943652 |
| LDL | overall | rs10018448,rs1260326,rs2977929,rs34894639,rs4801776,rs6499561 |
| logTG | overall | rs10018448,rs1128249,rs117643180,rs1260326,rs12974412,rs17096421,rs2943652,rs34894639,  rs36181536,rs4801776,rs5747934,rs72538440,rs7656569,rs77724138 |
| SBP | overall | rs10018448,rs1128249,rs117643180,rs17096421,rs2943652,rs36181536,rs4801776 |
| DBP | overall | rs10018448,rs1128249,rs117643180,rs17096421,rs2943652,rs36181536 |
| BMI | overall | rs10018448,rs1128249,rs1260326,rs2638315,rs34894639,rs6499561,rs7656569 |
| Glucose | Men | rs1128249,rs2943652,rs1260326 |
| Glucose | Women | rs1128249,rs1260326 |
| LDL | Men | rs10018448,rs1260326,rs34894639,rs4801776,rs6499561 |
| LDL | Women | rs10018448,rs1128249,rs1260326,rs2943652,rs34894639,rs4801776 |
| logTG | Men | rs10018448,rs1128249,rs117643180,rs1260326,rs17096421,rs2943652,rs34894639,rs36181536,  rs4801776,rs5747934,rs72538440,rs7656569 |
| logTG | Women | rs10018448,rs1128249,rs117643180,rs1260326,rs2943652,rs34894639,rs36181536,rs4801776,  rs5747934,rs72538440,rs7656569 |
| SBP | Men | rs2943652 |
| SBP | Women | rs117643180,rs4801776 |
| DBP | Men | No outlier |
| DBP | Women | rs36181536,rs5747934 |
| BMI | Men | rs2638315,rs370014171,rs1260326,rs34894639,rs7656569 |
| BMI | Women | rs2638315,rs1128249,rs34894639 |

SBP, systolic blood pressure; DBP, diastolic blood pressure; BMI, body mass index

Supplemental Table 10. Overall and sex-specific association of genetically predicted BCAAs with CVD risk factors using MR-Egger

| Outcome | Sex | Beta coefficient | 95% CI | *p* value | Intercept *p* value |
| --- | --- | --- | --- | --- | --- |
| Blood glucose | Overall | -0.05 | -0.28, 0.19 | 0.70 | 0.81 |
| LDL | Overall | 0.01 | -0.16, 0.18 | 0.92 | 0.51 |
| logTG | Overall | -0.03 | -0.74, 0.67 | 0.93 | 0.28 |
| Systolic blood pressure | Overall | 0.13 | -2.48, 2.75 | 0.92 | 0.16 |
| Diastolic blood pressure | Overall | -0.29 | -1.83, 1.24 | 0.71 | 0.11 |
| Body mass index | Overall | 0.03 | -0.17, 0.22 | 0.78 | 0.998 |
|  |  |  |  |  |  |
| Blood glucose | Men | -0.03 | -0.38, 0.32 | 0.87 | 0.72 |
| Blood glucose | Women | -0.08 | -0.33, 0.16 | 0.51 | 0.58 |
| LDL | Men | 0.02 | -0.19, 0.23 | 0.86 | 0.64 |
| LDL | Women | -0.05 | -0.37, 0.27 | 0.76 | 0.24 |
| logTG | Men | -0.10 | -0.81, 0.60 | 0.77 | 0.22 |
| logTG | Women | -0.10 | -0.93, 0.72 | 0.80 | 0.22 |
| Systolic blood pressure | Men | 0.07 | -0.09, 0.23 | 0.38 | 0.74 |
| Systolic blood pressure | Women | 0.04 | -0.13, 0.22 | 0.61 | 0.72 |
| Diastolic blood pressure | Men | 0.02 | -0.10, 0.15 | 0.71 | 0.62 |
| Diastolic blood pressure | Women | 0.04 | -0.14, 0.22 | 0.64 | 0.80 |
| Body mass index | Men | 0.14 | -0.09, 0.38 | 0.24 | 0.41 |
| Body mass index | Women | 0.07 | -0.14, 0.28 | 0.50 | 0.87 |

Supplemental Table 11. Genetic associations of 3-HIB with potential confounders

| Potential confounders | SNP | chr | EA | OA | Beta | SE | P value |
| --- | --- | --- | --- | --- | --- | --- | --- |
| Age completed full time education | rs10127775 | 1 | T | A | 0.004 | 0.002 | 7.12E-02 |
| Alcohol intake frequency | rs10127775 | 1 | T | A | -0.001 | 0.004 | 8.46E-01 |
| Current tobacco smoking | rs10127775 | 1 | T | A | 0.000 | 0.001 | 7.76E-01 |
| Processed meat intake | rs10127775 | 1 | T | A | 0.000 | 0.002 | 9.80E-01 |
| Townsend deprivation index at recruitment | rs10127775 | 1 | T | A | -0.007 | 0.002 | 6.16E-03 |
| Age completed full time education | rs117643180 | 17 | A | C | -0.015 | 0.008 | 4.81E-02 |
| Alcohol intake frequency | rs117643180 | 17 | A | C | 0.008 | 0.011 | 4.62E-01 |
| Current tobacco smoking | rs117643180 | 17 | A | C | 0.003 | 0.004 | 4.94E-01 |
| Processed meat intake | rs117643180 | 17 | A | C | 0.019 | 0.007 | 5.30E-03 |
| Townsend deprivation index at recruitment | rs117643180 | 17 | A | C | -0.002 | 0.007 | 7.92E-01 |
| Age completed full time education | rs12314700 | 12 | T | A | -0.003 | 0.003 | 2.94E-01 |
| Alcohol intake frequency | rs12314700 | 12 | T | A | -0.001 | 0.005 | 8.74E-01 |
| Current tobacco smoking | rs12314700 | 12 | T | A | 0.000 | 0.002 | 8.89E-01 |
| Processed meat intake | rs12314700 | 12 | T | A | 0.001 | 0.003 | 6.90E-01 |
| Townsend deprivation index at recruitment | rs12314700 | 12 | T | A | 0.000 | 0.003 | 9.64E-01 |
| Age completed full time education | rs2419604 | 10 | G | A | 0.004 | 0.003 | 1.11E-01 |
| Alcohol intake frequency | rs2419604 | 10 | G | A | 0.001 | 0.004 | 8.93E-01 |
| Current tobacco smoking | rs2419604 | 10 | G | A | 0.000 | 0.001 | 8.72E-01 |
| Processed meat intake | rs2419604 | 10 | G | A | 0.003 | 0.002 | 2.30E-01 |
| Townsend deprivation index at recruitment | rs2419604 | 10 | G | A | -0.004 | 0.003 | 1.77E-01 |
| Age completed full time education | rs2575876 | 9 | A | G | 0.001 | 0.003 | 6.70E-01 |
| Alcohol intake frequency | rs2575876 | 9 | A | G | -0.002 | 0.004 | 7.11E-01 |
| Current tobacco smoking | rs2575876 | 9 | A | G | 0.000 | 0.002 | 9.86E-01 |
| Processed meat intake | rs2575876 | 9 | A | G | 0.003 | 0.002 | 2.90E-01 |
| Townsend deprivation index at recruitment | rs2575876 | 9 | A | G | -0.001 | 0.003 | 6.82E-01 |
| Age completed full time education | rs2645433 | 8 | C | G | -0.001 | 0.003 | 5.53E-01 |
| Alcohol intake frequency | rs2645433 | 8 | C | G | 0.001 | 0.004 | 7.49E-01 |
| Current tobacco smoking | rs2645433 | 8 | C | G | 0.005 | 0.001 | 5.45E-04 |
| Processed meat intake | rs2645433 | 8 | C | G | -0.010 | 0.002 | 1.70E-06 |
| Townsend deprivation index at recruitment | rs2645433 | 8 | C | G | 0.003 | 0.002 | 1.63E-01 |
| Age completed full time education | rs28929474 | 14 | T | C | 0.022 | 0.009 | 1.20E-02 |
| Alcohol intake frequency | rs28929474 | 14 | T | C | 0.049 | 0.013 | 8.09E-05 |
| Current tobacco smoking | rs28929474 | 14 | T | C | -0.019 | 0.005 | 6.49E-05 |
| Processed meat intake | rs28929474 | 14 | T | C | 0.009 | 0.008 | 2.50E-01 |
| Townsend deprivation index at recruitment | rs28929474 | 14 | T | C | -0.016 | 0.008 | 5.24E-02 |
| Age completed full time education | rs35853021 | 15 | T | G | 0.006 | 0.003 | 2.11E-02 |
| Alcohol intake frequency | rs35853021 | 15 | T | G | -0.008 | 0.004 | 3.88E-02 |
| Current tobacco smoking | rs35853021 | 15 | T | G | -0.001 | 0.001 | 6.25E-01 |
| Processed meat intake | rs35853021 | 15 | T | G | -0.006 | 0.002 | 5.40E-03 |
| Townsend deprivation index at recruitment | rs35853021 | 15 | T | G | 0.005 | 0.002 | 3.20E-02 |
| Age completed full time education | rs4149307 | 9 | T | C | 0.001 | 0.003 | 6.75E-01 |
| Alcohol intake frequency | rs4149307 | 9 | T | C | -0.007 | 0.005 | 1.44E-01 |
| Current tobacco smoking | rs4149307 | 9 | T | C | 0.000 | 0.002 | 8.53E-01 |
| Processed meat intake | rs4149307 | 9 | T | C | 0.001 | 0.003 | 8.00E-01 |
| Townsend deprivation index at recruitment | rs4149307 | 9 | T | C | 0.002 | 0.003 | 4.83E-01 |
| Age completed full time education | rs429358 | 19 | C | T | 0.005 | 0.003 | 1.11E-01 |
| Alcohol intake frequency | rs429358 | 19 | C | T | -0.004 | 0.005 | 4.41E-01 |
| Current tobacco smoking | rs429358 | 19 | C | T | -0.006 | 0.002 | 7.71E-04 |
| Processed meat intake | rs429358 | 19 | C | T | -0.015 | 0.003 | 2.70E-07 |
| Townsend deprivation index at recruitment | rs429358 | 19 | C | T | -0.005 | 0.003 | 1.14E-01 |
| Age completed full time education | rs473224 | 15 | G | T | 0.004 | 0.003 | 2.24E-01 |
| Alcohol intake frequency | rs473224 | 15 | G | T | 0.009 | 0.005 | 7.33E-02 |
| Current tobacco smoking | rs473224 | 15 | G | T | -0.001 | 0.002 | 7.70E-01 |
| Processed meat intake | rs473224 | 15 | G | T | 0.004 | 0.003 | 1.60E-01 |
| Townsend deprivation index at recruitment | rs473224 | 15 | G | T | 0.003 | 0.003 | 3.13E-01 |
| Age completed full time education | rs585188 | 5 | A | G | 0.005 | 0.003 | 5.80E-02 |
| Alcohol intake frequency | rs585188 | 5 | A | G | 0.000 | 0.004 | 9.76E-01 |
| Current tobacco smoking | rs585188 | 5 | A | G | 0.000 | 0.002 | 9.23E-01 |
| Processed meat intake | rs585188 | 5 | A | G | -0.003 | 0.002 | 2.20E-01 |
| Townsend deprivation index at recruitment | rs585188 | 5 | A | G | -0.002 | 0.003 | 4.55E-01 |
| Age completed full time education | rs72702354 | 14 | G | A | 0.008 | 0.006 | 1.86E-01 |
| Alcohol intake frequency | rs72702354 | 14 | G | A | 0.024 | 0.009 | 7.10E-03 |
| Current tobacco smoking | rs72702354 | 14 | G | A | 0.001 | 0.003 | 6.99E-01 |
| Processed meat intake | rs72702354 | 14 | G | A | 0.000 | 0.005 | 9.50E-01 |
| Townsend deprivation index at recruitment | rs72702354 | 14 | G | A | 0.003 | 0.006 | 6.08E-01 |
| Age completed full time education | rs964184 | 11 | C | G | 0.000 | 0.004 | 9.21E-01 |
| Alcohol intake frequency | rs964184 | 11 | C | G | 0.003 | 0.005 | 5.58E-01 |
| Current tobacco smoking | rs964184 | 11 | C | G | 0.000 | 0.002 | 8.11E-01 |
| Processed meat intake | rs964184 | 11 | C | G | 0.005 | 0.003 | 9.70E-02 |
| Townsend deprivation index at recruitment | rs964184 | 11 | C | G | -0.003 | 0.003 | 4.10E-01 |
| Age completed full time education | rs9930957 | 16 | T | C | 0.002 | 0.003 | 4.93E-01 |
| Alcohol intake frequency | rs9930957 | 16 | T | C | -0.006 | 0.005 | 1.94E-01 |
| Current tobacco smoking | rs9930957 | 16 | T | C | 0.002 | 0.002 | 2.56E-01 |
| Processed meat intake | rs9930957 | 16 | T | C | 0.003 | 0.003 | 2.50E-01 |
| Townsend deprivation index at recruitment | rs9930957 | 16 | T | C | 0.004 | 0.003 | 2.13E-01 |
| Age completed full time education | rs9987289 | 8 | G | A | 0.004 | 0.004 | 3.45E-01 |
| Alcohol intake frequency | rs9987289 | 8 | G | A | -0.019 | 0.006 | 1.75E-03 |
| Current tobacco smoking | rs9987289 | 8 | G | A | -0.007 | 0.002 | 3.35E-03 |
| Processed meat intake | rs9987289 | 8 | G | A | -0.010 | 0.004 | 4.00E-03 |
| Townsend deprivation index at recruitment | rs9987289 | 8 | G | A | -0.001 | 0.004 | 8.66E-01 |

Supplemental Table 12. Overall and sex-specific association of genetically predicted 3-HIB with IHD and diabetes using MR-Egger

| Outcome | Sex | OR | 95% CI | *p* value | Intercept *p* value |
| --- | --- | --- | --- | --- | --- |
| IHD | Overall | 2.33 | 0.79, 6.83 | 0.12 | 0.37 |
| Diabetes | Overall | 0.87 | 0.36, 2.14 | 0.77 | 0.76 |
| IHD | men | 2.82 | 0.77, 10.36 | 0.12 | 0.41 |
| IHD | women | 1.54 | 0.63, 3.77 | 0.34 | 0.82 |
| Diabetes | men | 0.36 | 0.11, 1.12 | 0.08 | 0.12 |
| Diabetes | women | 0.78 | 0.21, 2.99 | 0.72 | 0.85 |

Supplemental Table 13. Outliers detected in MR-PRESSO in the overall and sex-specific associations of 3-HIB with CVD risk factors

| Outcome | Sex | Outliers |
| --- | --- | --- |
| Glucose | overall | rs2419604,rs9987289 |
| LDL | overall | rs10127775,rs1169297,rs117643180,rs2419604,rs2575876,rs2645433,rs28929474,rs35853021,  rs429358,rs473224,rs585188,rs72702354,rs964184,rs9930957,rs9987289 |
| logTG | overall | rs10127775,rs1169297,rs117643180,rs12314700,rs28929474,rs35853021,rs429358,rs473224,  rs585188,rs72702354,rs964184,rs9930957,rs9987289 |
| SBP | overall | rs117643180,rs2419604,rs2645433,rs28929474,rs429358,rs72702354,rs964184,rs9987289 |
| DBP | overall | rs117643180,rs2419604,rs2645433,rs28929474,rs964184,rs9987289 |
| BMI | overall | rs10127775,rs12314700,rs2419604,rs2645433,rs28929474,rs429358 |
| Glucose | Men | No outlier |
| Glucose | Women | rs9987289 |
| LDL | Men | rs1169297,rs2419604,rs2575876,rs28929474,rs35853021,rs429358,rs473224,rs585188,rs7270235,  rs964184,rs9930957,rs9987289 |
| LDL | Women | rs1169297,rs12314700,rs2419604,rs2575876,rs2645433,rs28929474,rs429358,rs473224,rs585188,  rs72702354,rs964184,rs9930957,rs9987289 |
| logTG | Men | rs10127775,rs12314700,rs35853021,rs429358,rs473224,rs585188,rs964184,rs9930957,rs9987289 |
| logTG | Women | rs10127775,rs1169297,rs12314700,rs2575876,rs35853021,rs429358,rs473224,rs585188,  rs72702354,rs964184,rs9930957,rs9987289 |
| SBP | Men | rs2419604,rs28929474,rs117643180,rs2645433 |
| SBP | Women | rs28929474,rs117643180,rs2645433,rs9987289 |
| DBP | Men | rs28929474,rs117643180,rs429358 |
| DBP | Women | rs28929474,rs117643180,rs2645433,rs9987289 |
| BMI | Men | rs28929474,rs429358,rs2645433 |
| BMI | Women | rs10127775,rs12314700,rs429358,rs2645433,rs2575876 |

SBP, systolic blood pressure; DBP, diastolic blood pressure; BMI, body mass index

Supplemental Table 14. Overall and sex-specific association of genetically predicted 3-HIB with CVD risk factors using MR-Egger

| Outcome | Sex | Beta coefficient | 95% CI | *p* value | Intercept *p* value |
| --- | --- | --- | --- | --- | --- |
| Blood glucose | Overall | -0.08 | -0.28, 0.12 | 0.43 | 0.50 |
| LDL | Overall | 0.46 | -1.05, 1.98 | 0.55 | 0.80 |
| logTG | Overall | 0.39 | -1.49, 2.27 | 0.68 | 0.93 |
| systolic blood pressure | Overall | 4.92 | 1.70, 8.14 | 0.003 | 0.004 |
| diastolic blood pressure | Overall | 1.69 | -0.29, 3.66 | 0.09 | 0.07 |
| Body mass index (BMI) | Overall | 0.04 | -0.26, 0.34 | 0.79 | 0.80 |
|  |  |  |  |  |  |
| Blood glucose | men | -0.30 | -0.60, -0.01 | 0.04 | 0.22 |
| Blood glucose | women | -0.24 | -0.46, -0.02 | 0.03 | 0.08 |
| LDL | men | 0.74 | -1.03, 2.50 | 0.41 | 0.69 |
| LDL | women | 0.19 | -2.41, 2.79 | 0.89 | 0.79 |
| logTG | men | 0.09 | -2.26, 2.44 | 0.94 | 0.58 |
| logTG | women | 0.26 | -2.01, 2.53 | 0.82 | 0.80 |
| systolic blood pressure | men | 0.45 | 0.18, 0.72 | 0.00 | 0.0004 |
| systolic blood pressure | women | 0.19 | -0.10, 0.48 | 0.20 | 0.27 |
| diastolic blood pressure | men | 0.28 | -0.01, 0.58 | 0.06 | 0.01 |
| diastolic blood pressure | women | 0.05 | -0.30, 0.41 | 0.77 | 0.77 |
| Body mass index (BMI) | men | 0.23 | -0.09, 0.55 | 0.17 | 0.11 |
| Body mass index (BMI) | women | 0.20 | -0.20, 0.60 | 0.33 | 0.43 |

Supplemental Table 15. Power calculation

| Exposure | Outcome | Detectable OR at 80% power  (in men and women) | Detectable OR at 80% power  (in men) | Detectable OR at 80% power  (in women) |
| --- | --- | --- | --- | --- |
| BCAAs | IHD | 0.93/1.08 | 0.85/1.18 | 0.81/1.24 |
| BCAAs | Diabetes | 0.92/1.09 | 0.87/1.14 | 0.86/1.17 |
| 3-HIB | IHD | 0.92/1.09 | 0.83/1.21 | 0.78/1.29 |
| 3-HIB | Diabetes | 0.91/1.10 | 0.85/1.17 | 0.83/1.20 |
|  |  |  |  |  |
|  |  | Beta-coefficient  (in men and women) | Beta-coefficient  (in men) | Beta-coefficient  (in women) |
| BCAAs | Glucose | 0.053 | 0.054 | 0.059 |
|  | Lipids | 0.026 | 0.029 | 0.032 |
|  | Blood pressure | 0.028 | 0.054 | 0.059 |
|  | BMI | 0.035 | 0.054 | 0.059 |
| 3-HIB | Glucose | 0.063 | 0.064 | 0.069 |
|  | Lipids | 0.031 | 0.034 | 0.037 |
|  | Blood pressure | 0.032 | 0.064 | 0.069 |
|  | BMI | 0.041 | 0.064 | 0.069 |

The R^2^ was calculated as beta^2^ * 2 * MAF * (1-MAF), where beta is the genetic association with the exposure and MAF is the minor allele frequency of the genetic variant. The R^2^ was 0.014 for overall and sex-specific associations of BCAA, 0.01 for overall association and sex-specific associations of HIB.

Supplemental Table 16. Associations of genetically predicted BCAAs with body mass index using different analytic methods

| Sex | Methods | Beta coefficient | 95% CI | P value |
| --- | --- | --- | --- | --- |
| Overall | IVW | 0.03 | -0.06, 0.12 | 0.56 |
|  | Weighted median | 0.07 | 0.03, 0.11 | 0.0007 |
|  | Weighted mode | 0.07 | 0.03, 0.11 | 0.0004 |
|  | MR-PRESSO | 0.04 | -0.05, 0.13 | 0.35 |
| Men | IVW | 0.05 | -0.05, 0.16 | 0.33 |
|  | Weighted median | 0.08 | 0.02, 0.14 | 0.007 |
|  | Weighted mode | 0.11 | 0.04, 0.17 | 0.0007 |
|  | MR-PRESSO | 0.05 | -0.02, 0.13 | 0.17 |
| Women | IVW | 0.06 | -0.04, 0.15 | 0.23 |
|  | Weighted median | 0.09 | 0.03, 0.15 | 0.005 |
|  | Weighted mode | 0.10 | 0.04, 0.16 | 0.002 |
|  | MR-PRESSO | 0.09 | 0.04, 0.15 | 0.002 |

Supplemental Figure 1. Scatter plot on the association of genetically predicted BCAAs and 3-HIB with ischemic heart disease (IHD) and diabetes. (a) BCAAs and IHD; (b) BCAAs and diabetes; (c) 3-HIB and IHD; (d) 3-HIB and diabetes.


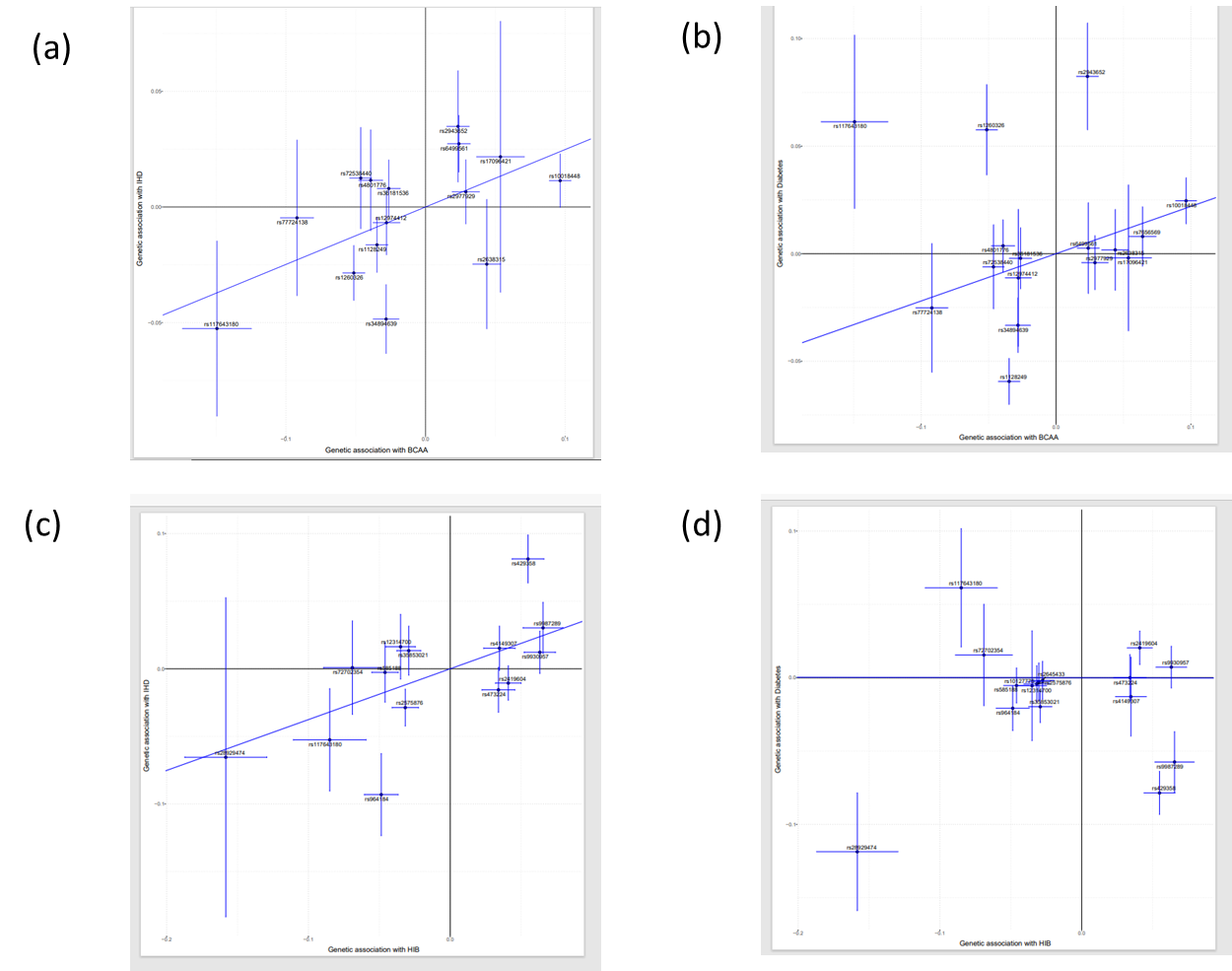


Supplemental Figure 2. Sensitivity analysis on the overall associations of genetically predicted BCAAs with IHD and diabetes after excluding the pleiotropic SNP (rs1260326)


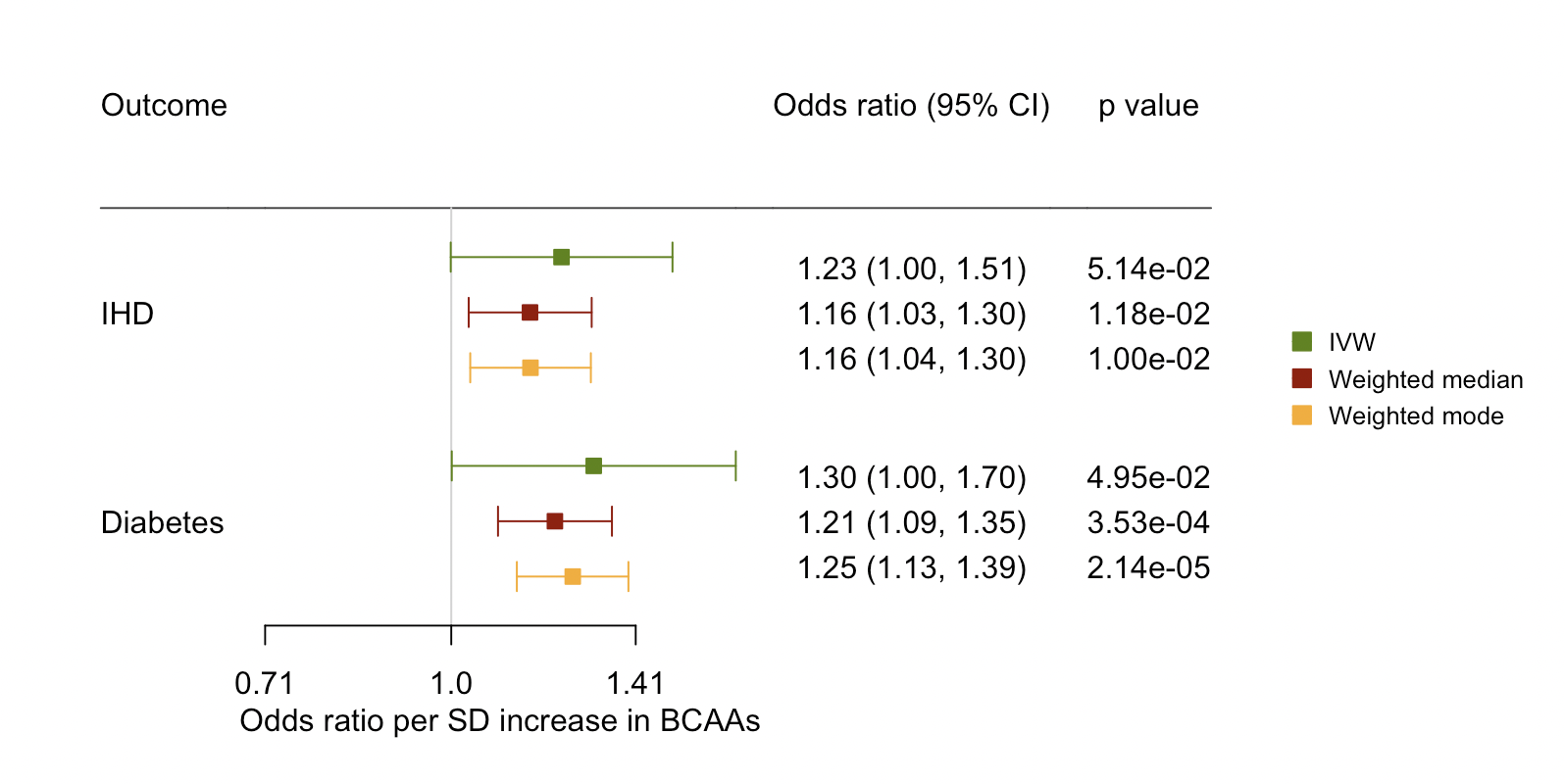


Supplemental Figure 3. Sensitivity analysis on the sex-specific associations of genetically predicted BCAAs with IHD and diabetes in the UK Biobank after excluding the pleiotropic SNP (rs1260326)


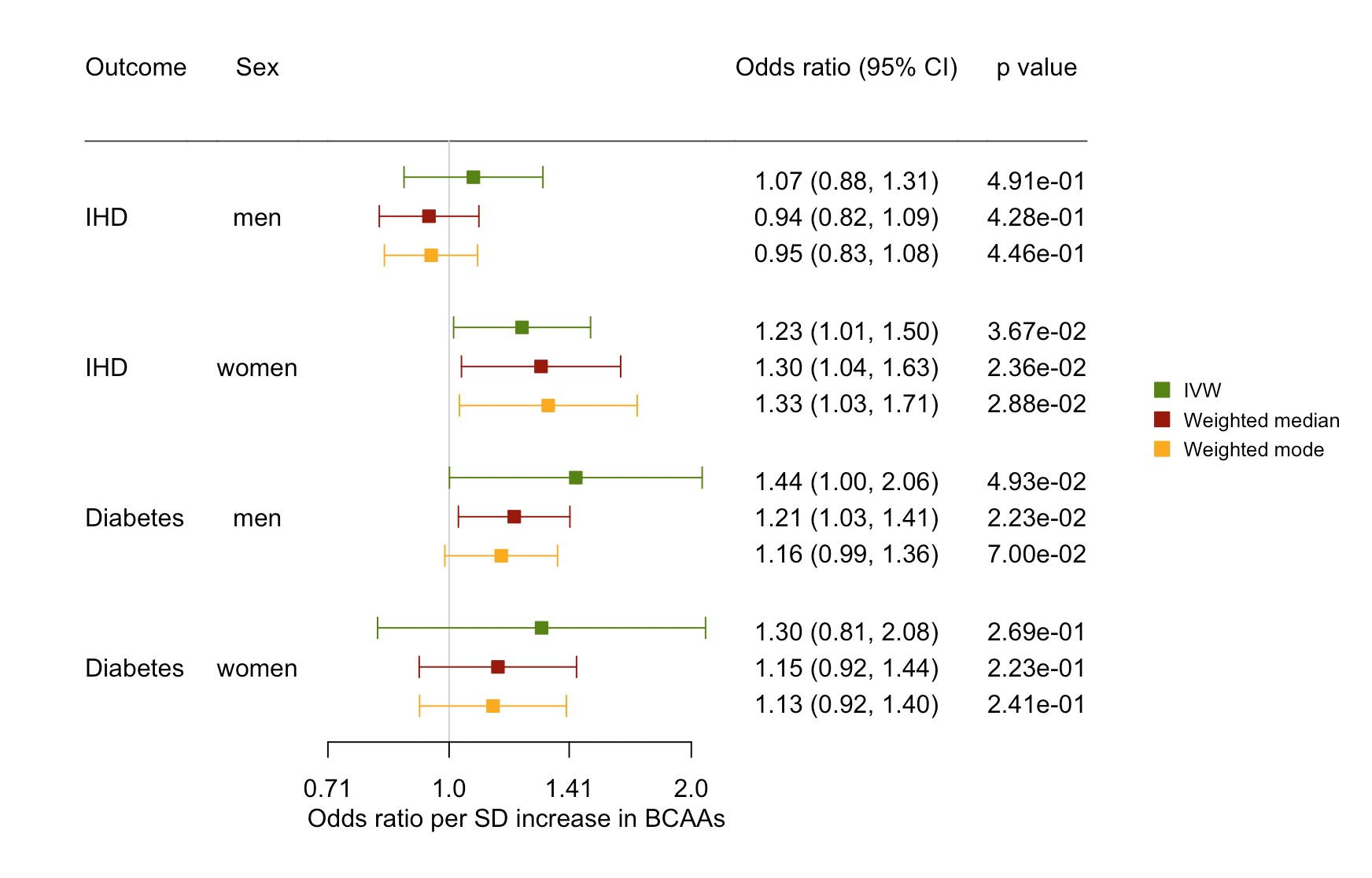


Supplemental Figure 4. Associations of genetically predicted BCAAs with cardiovascular disease risk factors using different analytic methods


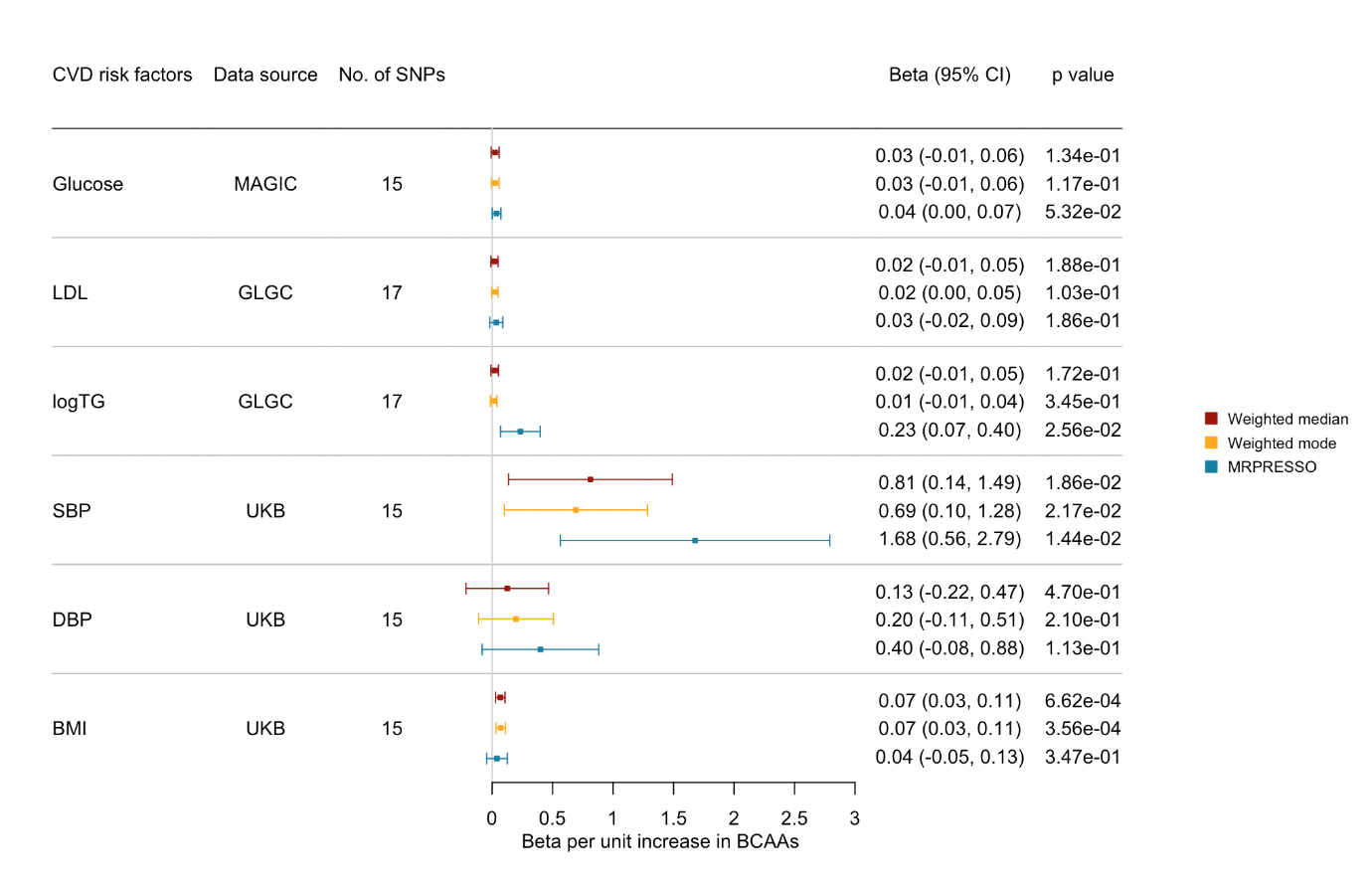


Supplemental Figure 5. Sex-specific associations of genetically predicted BCAAs with cardiovascular disease risk factors using different analytic methods in GLGC (for lipids) and the UK Biobank (for other risk factors)


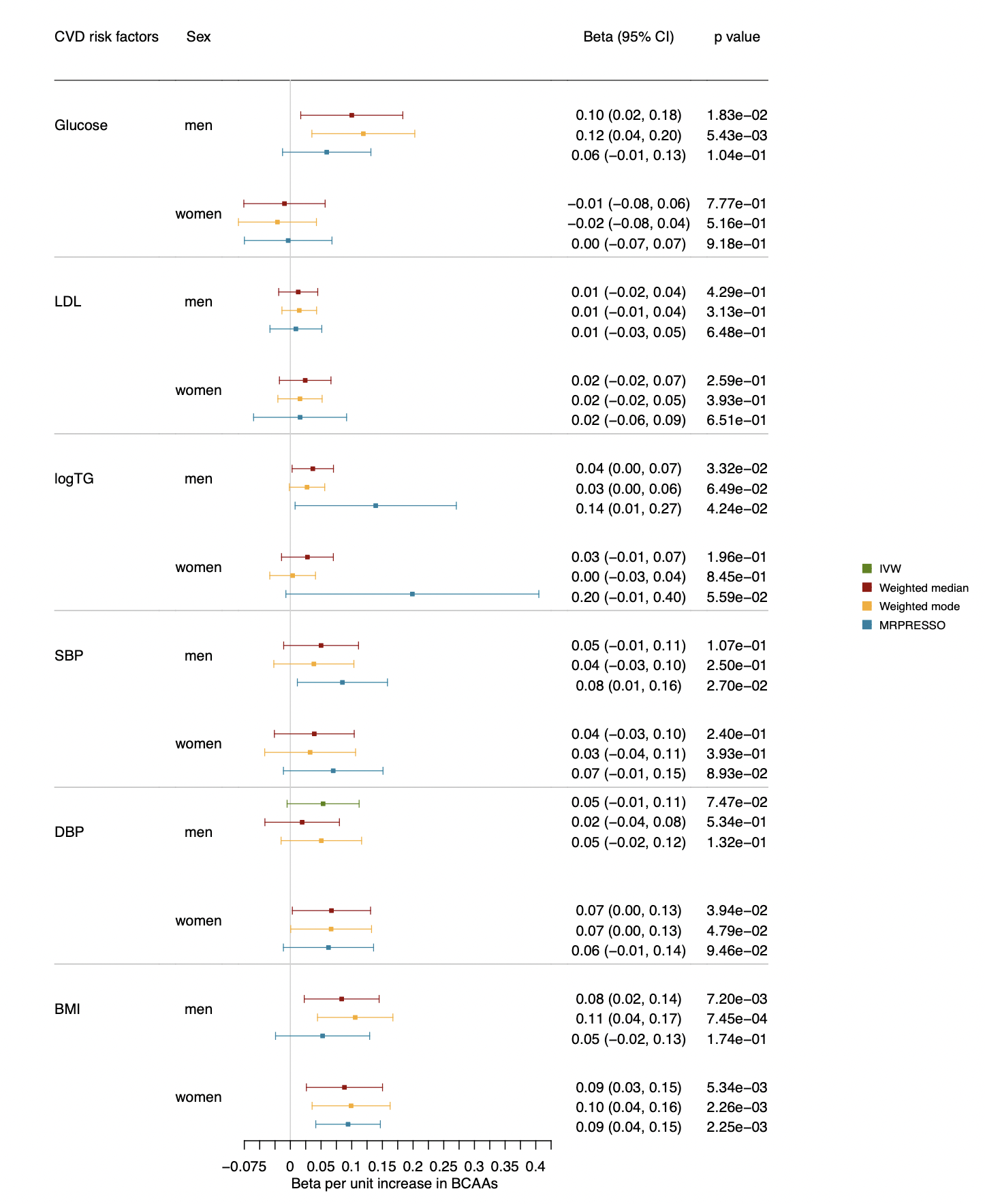


Supplemental Figure 6. Associations of genetically predicted 3-HIB with cardiovascular disease risk factors using different analytic methods


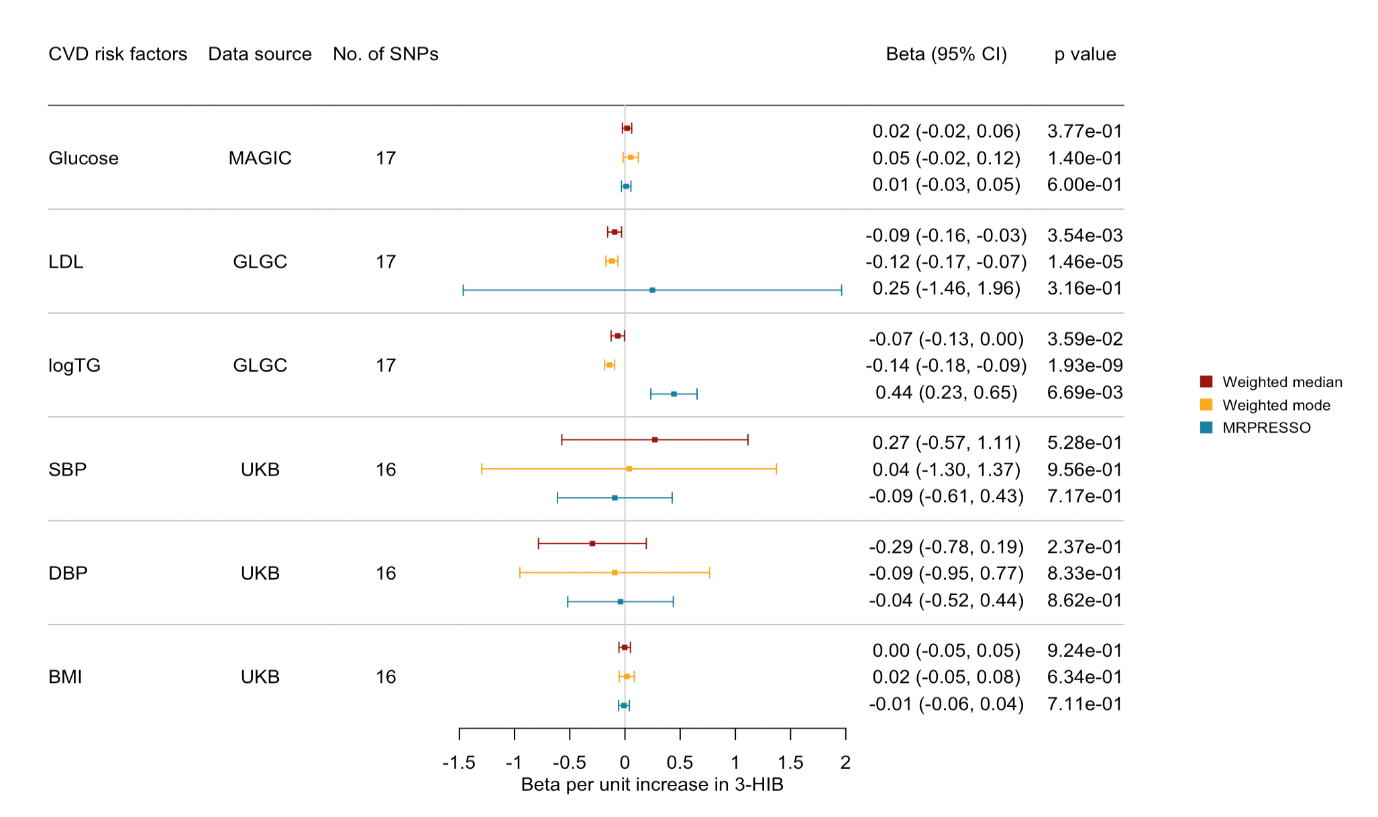


Supplemental Figure 7. Sex-specific associations of genetically predicted 3-HIB with cardiovascular disease risk factors using different analytic methods in GLGC (for lipids) and the UK Biobank (for other risk factors)


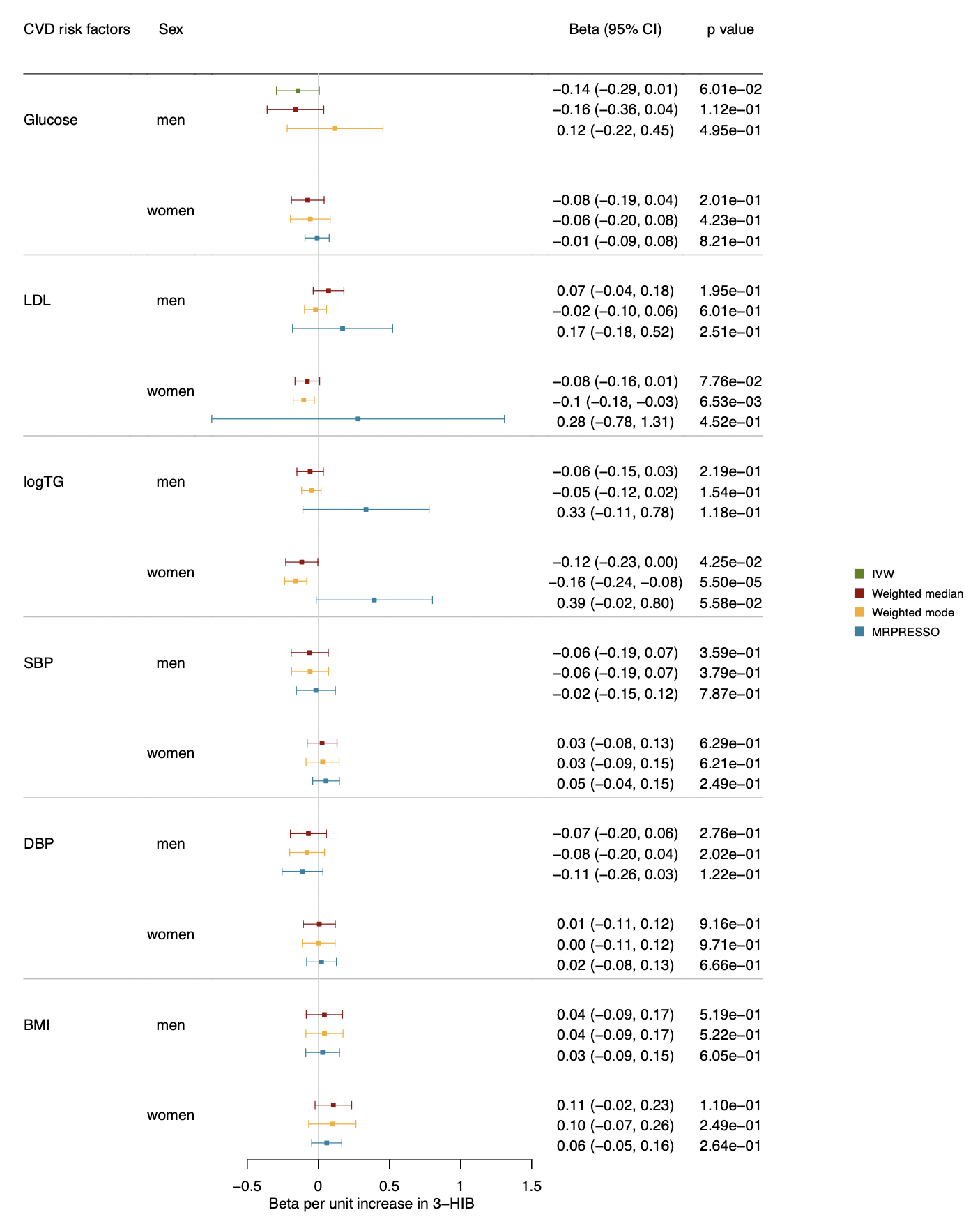

Supplement: Supplementary material [file EMS187386-supplement-Supplementary_material.docx]
